# Facilitating collaboration between public health researchers and policymakers: a scoping review of global practices, barriers and facilitators

**DOI:** 10.1186/s12961-026-01443-y

**Published:** 2026-01-21

**Authors:** Alaa Hussain Subahe, Philip Baker, Remco Polman

**Affiliations:** 1 School of Public Health and Social Work, Queensland University of Technology, Victoria Park Rd, Kelvin Grove, QLD 4059 Australia; 2 Faculty of Nursing, Umm Al Qura University, Makkah, Saudi Arabia; 3 Australian Centre for Health Law Research and School of Public Health and Social Work, Queensland University of Technology, Victoria Park Rd, Kelvin Grove, QLD 4059 Australia; 4 Graduate School of Public, Health Yonsei University, Seoul, South Korea; 5 Faculty of Medicine, UiTM, Puncak Alam, Selangor Malaysia; 6 School of Exercise and Nutrition Science, Department of Health and PE, Queensland University of Technology, Brisbane, Australia; 7 The Education University of Hong Kong, Ting Kok, China

**Keywords:** Research-policy collaboration, Co-production of knowledge, Capacity building, Public health policy, Evidence-informed decision-making, Knowledge translation

## Abstract

**Background:**

Effective collaboration between public health researchers and policymakers is critical for translating evidence into actionable policies. However, such collaboration is often undermined by misaligned priorities, structural barriers, and fragmented systems. This scoping review synthesizes global evidence on the strategies, facilitators and barriers shaping these collaborations, with a focus on how they enhance the uptake and use of research in policymaking.

**Methods:**

A scoping review was conducted using a systematic search across four electronic databases (SCOPUS, Embase, Web of Science and Medline via PubMed), targeting studies published between 2019 and 2024. Eligible studies discussed strategies, barriers and facilitators for collaboration between researchers and policymakers in public health. Data were extracted on study context, collaboration mechanisms, enabling conditions and reported impacts.

**Results:**

A total of 24 studies were included, revealing four interdependent strategies for effective collaboration. Most notably, co-production of knowledge emerged in two distinct forms: as a deliberately structured intervention and as an emergent outcome of sustained engagement over time. This dual nature of co-production offers a critical lens for understanding how collaboration evolves in practice. Other key strategies included capacity building at both individual and organizational levels, the use of structured communication and feedback mechanisms to align research with policy needs, and the establishment of governance frameworks to institutionalize research–policy partnerships. Collaborations that were formalized, embedded within institutions, and maintained through iterative engagement were more likely to result in increased research uptake and enduring policy impact.

**Conclusions:**

These findings underscore that structured collaboration, particularly when supported by institutional mechanisms, ongoing capacity building and iterative engagement, plays a critical role in increasing the use of research in public health policymaking. By distinguishing co-production as both a deliberate strategy and an emergent outcome, this review offers new insight into how collaborative models can be designed or evolved to strengthen evidence-informed decision-making.

**Supplementary Information:**

The online version contains supplementary material available at 10.1186/s12961-026-01443-y.

## Background

In recent years, there has been a growing emphasis on evidence-informed decision-making (EIDM) as a fundamental principle for improving public health policies and interventions [35]. The integration of research evidence into policymaking has been associated with more effective and efficient public health programs, ensuring that decisions are responsive to evolving and complex health challenges [53]. However, despite the well-documented benefits of incorporating research into policy, significant challenges persist in translating scientific knowledge into actionable public health policies [4, 32].

One of the key obstacles to research utilization in policymaking is the fragmented nature of researcher-policymaker collaboration. Traditional models have often conceptualized this relationship as a linear knowledge transfer process, where evidence is “pushed” from academia into policy systems. However, policymaking is a dynamic and iterative process influenced by political contexts, institutional frameworks and shifting timelines. These factors shape how evidence is interpreted and applied [6]. These contextual complexities underscore the limitations of linear knowledge transfer models and highlight the need for more adaptive, relational and embedded approaches.

Efforts to strengthen collaboration have been impeded by a variety of systemic and structural barriers. These include misaligned incentives between academia and government, limited research literacy among policymakers and communication gaps that constrain mutual understanding [15]. Moreover, policymaking environments are often decentralized and politically contested, where scientific evidence must compete with practitioner experience, electoral pressures, ideological values and institutional inertia [2, 5]. In this context, The European Commission’s Joint Research Centre (JRC) has called for moving beyond reductive notions of “barriers” and instead developing robust knowledge infrastructures and capabilities that facilitate strategic engagement between researchers and policymakers [44]. These include synthesizing complex evidence, managing networks of expertise, and reframing research to align with policymaker needs and political timing [45].

In light of these systemic challenges, there has been increasing interest in developing more collaborative models of engagement between researchers and decision-makers to close the persistent gap between knowledge production and policy action. A range of approaches such as integrated knowledge translation (iKT), partnerships, embedded roles and co-creation models have emerged across diverse contexts to support this goal [15, 30]. However, despite these developments, integrating research into policymaking in a sustained and institutionalized manner remains complex. Most initiatives are limited to individual projects or short-term activities, with relatively few examples of mechanisms that support long-term interaction, mutual learning or embedded collaboration structures [18, 25, 38].

Over the past decade, several reviews have explored the dynamics of researcher–policymaker partnerships in public health and healthcare. McIsaac and Riley [25] identified key co-production actions in engaged scholarship but offered limited insight into how these practices are embedded institutionally. Gagliardi et al. [11] reviewed iKT models in healthcare, highlighting its potential to foster collaborative decision-making while noting variability in implementation and a lack of theoretical consistency. Hoekstra et al. [15] synthesized models of research-policy interaction across sectors, emphasizing that successful collaboration depends heavily on context-specific adaptation. More recently, Kneale et al. [18] mapped embedded researcher interventions, identifying different forms of collaboration but offering limited insight into how these roles are operationalized in practice or sustained over time. Similarly, Voller et al. [50] reviewed partnership guidelines for global research collaboration yet found a persistent gap between normative frameworks and sustained, real-world implementation. While these studies have advanced our understanding of researcher–policymaker partnerships by offering valuable conceptual frameworks and typologies, questions remain about how such collaborations function in practice and what enables them to support the use of evidence in policymaking.

In response, this scoping review synthesizes global literature on collaboration between public health researchers and policymakers, with a focus on how such partnerships operate and what factors support their effectiveness. It explores both the practical strategies that enable collaboration and the ways in which these strategies support the use of research evidence in policymaking.

## Methods

The review was guided by the scoping review methodological framework established by Arksey and O’Malley [1]. The review followed the five key steps outlined in this framework: identifying the research question, conducting a comprehensive literature search, selecting relevant studies, extracting and charting the data and synthesising and reporting the results. This methodological approach is particularly suitable for assessing the wide-ranging literature on complex issues [37] which, in this case, involves understanding the dynamics of collaborative practices in public health policy development. This approach allows for comprehensive insights into the complexities of effective collaboration, helping to identify and address knowledge gaps and provide guidance for policymakers and researchers on improving joint efforts over time. The review process involved a detailed and systematic literature search to ensure a thorough exploration of the existing evidence.

Four electronic databases (SCOPUS, Embase, Web of Science and Medline via PubMed) were utilized for the search, using Boolean operators with specific keywords. The primary search terms included combinations of the following keywords: (“Knowledge Translation” OR “Knowledge Transfer” OR “Knowledge Exchange” OR “Knowledge Utilization” OR “Knowledge Mobilization” OR “Knowledge Integration” OR “Knowledge Dissemination” OR “Knowledge Implementation” OR “Research Translation” OR “Research Utilization” OR “Knowledge Application” OR “Information Translation” OR “Translational Science” OR “Research-to-Practice” OR “Knowledge Conversion” OR “Knowledge Transfer and Exchange” OR “Research Communication” OR “Integrated Knowledge translation” OR “Integrated Knowledge Transfer” OR “partnership research” OR “Participatory Approach” OR “Collaborative Research” OR “Co-Production of Knowledge”) AND (“Policymakers” OR “Decision Makers” OR “Decision-Makers” OR “Stakeholders” OR “Government officials”) AND (“Researchers” OR “Academics” OR “Academia” OR “Scholar” OR “Scientists”) AND (“Strategy” OR “Strategies” OR “Practice” OR “Model”) AND (“Public Health”). The search focused on articles published in English between 2019 and 2024. This time frame was selected to capture a period marked by intensified attention to evidence-informed policymaking, particularly in response to the COVID-19 pandemic. The global health crisis amplified the urgency of timely, policy-relevant research and catalysed new forms of collaboration between researchers and policymakers. During this period, existing approaches such as digital evidence platforms, policy labs and iKT strategies were adapted and applied more broadly. International initiatives, such as the revitalization of the WHO’s Evidence-Informed Policy Network (EVIPNet) [52] and the UN’s Research Roadmap for COVID-19 Recovery [47], reflected this momentum toward stronger research–policy linkages. Focusing the review on this contemporary window allows for the synthesis of collaboration strategies that emerged or evolved under these high-pressure, real-world conditions without conflating them with earlier, often more fragmented approaches.

This review included studies that explicitly discussed strategies, interventions, models or frameworks to facilitate collaboration between public health researchers and policymakers. It also considered studies that identified barriers and facilitators to such collaborations, particularly in relation to how they influence the use of research evidence in public health decision-making. These studies were conducted in diverse contexts, including global, regional and national settings, to provide insights into successful practices in public health research–policy collaboration. The inclusion criteria were broad in terms of study design, capturing a wide spectrum of evidence through qualitative, quantitative, mixed-methods studies and case studies. In addition, the studies selected reported on outcomes related to the implementation of research evidence in public health policy, which included but were not limited to policy changes, improved health outcomes, increased research utilization and enhanced researcher–policymaker collaboration.

Studies focused on clinical research rather than public health were excluded, as they do not align with the scope of this review, which targets public health initiatives. Studies that did not involve policymakers were excluded, as the review specifically examines interactions between researchers and public health decision-makers. In addition, studies addressing only collaboration between researchers and the community, without considering the role of policymakers or public health strategies, were excluded.

**Fig. 1:**
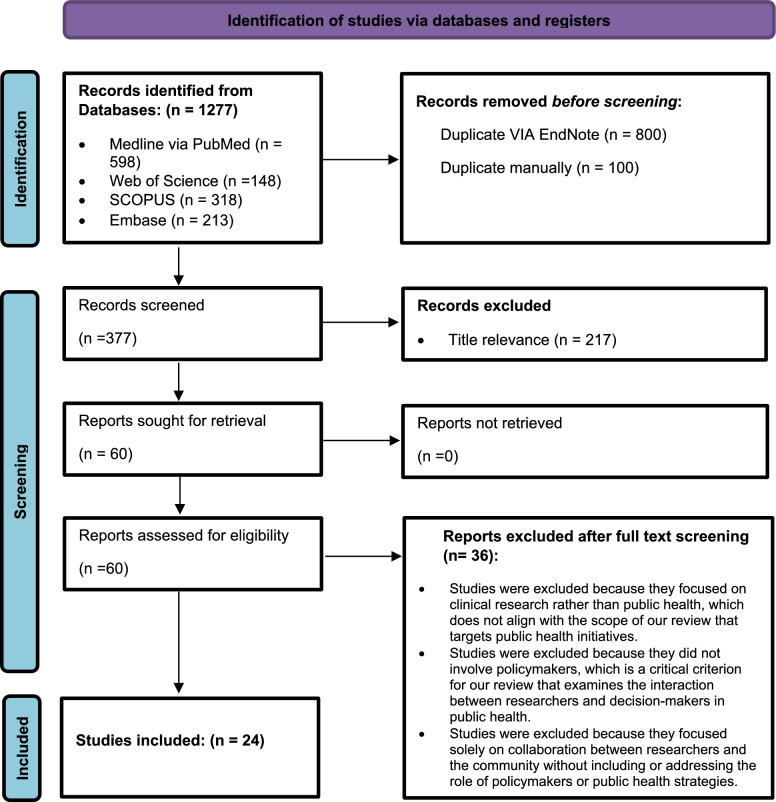
PRISMA flowchart showing literature search and article selection for the scoping review

Duplicate records were removed using EndNote 20 and manual checking. Titles, abstracts and full texts were screened independently by two reviewers according to the eligibility criteria, with discrepancies resolved through discussion and consensus. Ultimately, 24 studies were included in the final analysis. The literature search and selection process are detailed in the PRISMA flow diagram (Fig. 1).

Data extraction was also conducted independently by two reviewers using a predefined template (see Table 1 in Appendix 1), with any discrepancies similarly resolved through discussion. The extraction template was piloted on three studies to refine the categories and ensure consistency. Extracted variables included study population, research design, collaboration strategies, barriers and facilitators to research-policy integration and reported outcomes. This structure ensured a comprehensive and consistent collection of data across studies, allowing for a systematic comparison of collaboration strategies and their effectiveness in integrating research into policy and practice.

**Table 1:** Barriers and facilitators to research–policy engagement identified in the scoping review

Theme	Barriers	Facilitators
Capacity and infrastructure	Lack of funding, insufficient human capital, inadequate institutional infrastructure	Training programs, capacity-building workshops, and embedded research units
Alignment and prioritization	Misaligned goals, timelines and incentives between researchers and policymakers	Early stakeholder involvement, joint agenda-setting and co-production frameworks
Communication and translation	Technical language, inconsistent messaging, difficulty interpreting findings	Digital platforms, policy briefs, structured feedback loops and translation specialists
Governance and institutionalization	Bureaucratic delays, rigid structures and unclear roles	Advisory groups, co-leadership structures and liaison roles within institutions
Trust and relationships	High stakeholder turnover, transactional interactions and weak continuity	Long-term engagement, mutual respect and role clarity and continuity
Cultural and political context	Resistance to evidence use, skepticism of external research and policy inertia	Integration of Indigenous and local leadership and cultural sensitivity in framing
Engagement structures	Lack of platforms for engagement and logistical and coordination challenges	Health Economics and Policy Units (HEPUs), integrated Knowledge Translation (KT) networks and multi-level forums

An inductive thematic synthesis approach was employed to analyse the extracted data. Themes were identified through iterative coding and collaborative discussion among the research team. In line with scoping review methodology, no formal quality appraisal was conducted. This decision reflects the exploratory nature of the review but may limit the interpretation of study rigour. Grey literature was excluded to ensure methodological rigour and comparability across studies. The review focused on peer-reviewed publications.

## Results

A total of 24 studies were included in this scoping review, covering diverse methodological approaches: qualitative (*n* = 5), quantitative (*n* = 2), mixed-methods (*n* = 3) and case studies (*n* = 5). The remaining studies (*n* = 9) primarily examined the implementation and evaluation of structured collaboration approaches rather than following a specific research design. These studies were geographically diverse, spanning high-income countries such as Australia, Canada, the United States and Europe, as well as low- and middle-income countries (LMICs) across Africa, Latin America and other regions.

### Collaboration strategies

The review identified four interdependent strategies supporting effective collaboration between researchers and policymakers: co-production of knowledge, capacity building, structured communication and feedback and institutional governance mechanisms. These strategies did not operate in isolation but rather reinforced one another, forming a systemic approach to embedding research within policy processes. Details of each study and the specific strategies employed are provided in the extracted table in Appendix 1.

- ***Co-Production of Knowledge***

Co-production of knowledge emerged as both a deliberate strategy and an emergent outcome. In 12 studies [8, 13, 14, 17, 23, 26, 31, 36, 39, 40, 46, 51], co-production was intentionally designed and operationalized through structured mechanisms. These included embedded research units, policy fellowships, co-governance committees and institutionalized platforms that formalized collaboration between researchers and decision-makers. For instance, Haynes et al. [13] embedded policy fellows within state public health agencies in Australia, fostering hybrid roles that enabled researchers to directly shape health policy agendas. Pearce et al. [36] described a Health Policy Partnership Unit that coordinated structured feedback loops and co-produced evidence briefs with ministry stakeholders. Nguyen et al. [31] established formal national immunization coordination platforms, integrating researchers into real-time policy cycles. Similarly, Sibbald et al. [40] applied an iKT model using co-chaired advisory panels to set priorities and review outputs whereas Heenan et al. [14] used project charters and formal engagement agreements to ensure sustained collaboration with mental health policy actors throughout the research process. Mendell and Richardson [26], and Williamson et al. [51], embedded shared governance structures that linked research activities to program evaluation and policy oversight functions. These intervention-based approaches enabled co-production to become a repeatable governance function, often supported by dedicated funding, clear role definitions and institutional mandates.

In contrast, 12 studies [9, 21–24, 26–28, 41, 46, 48, 49] found that co-production emerged organically rather than being explicitly designed as an intervention. These studies observed that sustained engagement between researchers and stakeholders over time led to co-production practices developing naturally. The long-term interactions between researchers and policy actors created opportunities for collaboration that were not initially planned but developed as mutual trust and shared goals evolved. Dunn et al. [9] documented how researchers gradually became embedded in policy teams through consistent informal engagement, eventually participating in strategic discussions without designated roles. Smith et al. [41] described how sustained involvement in a violence prevention initiative led to researchers being trusted as informal advisors to local government. Valaitis et al. [48] observed that routine presence in planning meetings created embedded researcher roles that were never formally institutionalized but were functionally indispensable. Laird et al. [21] and Leslie et al. [22] noted that long-term participation in annual knowledge-sharing events fostered co-production by deepening trust and shared understanding. In similar fashion, Nabyonga-Orem et al. [28] reported how researchers in several African countries evolved into trusted policy advisors through adaptive, responsive engagement, rather than formal appointment. Finally, Mpando [27] illustrated how peer mentorship networks within Malawi’s Ministry of Health gradually brought researchers into implementation dialogues without the need for structural embedding.

- ***Capacity Building***

Capacity building was identified as a key strategy identified in 12 studies [9, 10, 13, 16, 17, 26, 28, 30, 31, 36, 41, 46, 51], aimed at strengthening the abilities of individuals and organisations to effectively generate, interpret, and apply research evidence within policymaking contexts. These studies consistently highlighted the dual focus of capacity-building efforts: enhancing individual competencies and developing institutional infrastructures that support sustained research–policy engagement.

Individual capacity building was evident in nine studies [9, 10, 13, 16, 17, 26, 28, 30, 31, 36], which focused on equipping policymakers, practitioners and researchers with the necessary skills to interpret and apply research findings effectively. These studies implemented tailored training programs, workshops and mentorship initiatives. For example, Dunn et al. [9] and Fulone et al. [10] highlighted the role of interactive workshops in strengthening evidence-based decision-making among policymakers, while Jessani et al. [16] and Kalibala and Nutley [17] described mentorship and experiential learning models that promoted cross-sectoral collaboration. Similarly, Haynes et al. [13] and Nguyen et al. [30, 31] reported on structured training interventions designed to enhance research literacy and critical engagement with evidence among decision-makers. Collectively, these efforts supported the development of a workforce capable of engaging meaningfully in knowledge translation and evidence-informed policymaking.

Organizational capacity building was highlighted in five studies [13, 26, 28, 41, 51], which emphasized the creation of institutional frameworks to support long-term research-policy collaborations. These included the establishment of embedded research units, policy advisory groups and formalized knowledge exchange mechanisms. Williamson et al. [51] and Smith et al. [41] detailed how policy research units within government institutions facilitated sustained collaboration between researchers and policymakers. Similarly, Haynes et al. [13] and Nabyonga-Orem et al. [28] found that structured governance arrangements improved the consistency and depth of research use in policy. Furthermore, Mendell et al. [26] examined the role of knowledge brokers in strengthening organizational capacity by serving as intermediaries between research institutions and policy entities, ensuring that research evidence was continuously adapted to evolving policy needs. These institutional mechanisms provided the structural support necessary for sustained, systematic and scalable research–policy engagement.

- ***Structured communication and feedback mechanisms***

Structured communication and feedback mechanisms were identified as critical enablers of research–policy collaboration in 10 studies [9, 22, 26, 28, 30, 31, 36, 41, 46, 49, 51]. These mechanisms facilitated consistent dialogue, enabled timely exchange of knowledge and ensured that research remained aligned with evolving policy demands. Across these studies, structured communication included formal meetings, digital engagement platforms and iterative feedback systems, all of which played a significant role in reinforcing mutual understanding and sustaining collaborative momentum.

Structured meetings and workshops were widely used to promote direct and sustained engagement. Williamson et al. [51] and Towfighi et al. [46] found that regular stakeholder workshops provided opportunities for researchers and policymakers to discuss key research findings, align priorities and collaboratively refine research objectives on the basis of policy needs. Similarly, Leslie et al. [22] emphasized the role of virtual communication platforms in overcoming logistical and geographical barriers, particularly in multi-country collaborations, where digital platforms facilitated real-time interactions and dynamic knowledge sharing.

Feedback mechanisms were also central to ensuring policy relevance throughout the research process. Nguyen et al. [30, 31] and Mendell et al. [26] documented the effectiveness of iterative feedback loops, which allowed for research adjustments based on policymakers’ evolving needs and priorities. Pearce et al. [36] and Smith et al. [41] further demonstrated how digital feedback tools improved accessibility and usability of research, fostering a two-way flow of knowledge and enhancing the practical application of evidence in policy settings. Collectively, these studies underscored that communication infrastructure was not merely a logistical support, but a core element of effective, adaptive and policy-responsive research collaboration.

- ***Institutional governance mechanisms***

Institutional governance mechanisms were identified in seven studies [9, 13, 28, 30, 31, 36, 41, 51] as foundational for sustaining research–policy partnerships over time. These mechanisms included co-leadership arrangements, formal advisory panels, steering committees, and structured partnership agreements, all of which embedded shared authority and accountability within institutional frameworks. For example, Nguyen et al. [30, 31] described the integration of policymakers into governance bodies that oversaw research direction and ensured alignment with policy agendas, while Smith et al. [41] and Dunn et al. [9] reported how institutional steering committees facilitated coordination and continuity despite political and personnel turnover. Pearce et al. [36] emphasized the value of codified decision-making processes in enhancing legitimacy and transparency across multisectoral collaborations. Similarly, the Australian Prevention Partnership Centre [13] and the Thanzi Programme in East Africa [28] institutionalized collaboration through embedded structures that enabled adaptive policy engagement, legislative uptake and programmatic scaling. These studies illustrate that robust governance systems do more than sustain partnerships – they transform them into durable, embedded mechanisms for evidence-informed policymaking.

### Barriers to effective collaboration

Several studies identified persistent challenges to effective collaboration between researchers and policymakers. One major barrier was resource constraints, particularly the lack of financial and human capital required to support sustained engagement [16, 21, 28, 39, 40]. Inadequate funding and institutional infrastructure often limited the ability to maintain long-term research–policy partnerships.

Another recurrent challenge was misalignment of priorities. Studies reported tension between the timelines, incentives and goals of researchers and policymakers. While policymakers tended to prioritize urgent political agendas, researchers often focused on longer-term academic outputs [9, 14, 51]. These divergent pressures frequently delayed or diluted the integration of research into policy.

Communication gaps also posed a significant barrier, with studies reporting inconsistent messaging between stakeholders, leading to misunderstandings and delays in translating research into action [10, 13, 43]. Some studies noted that policymakers struggled to interpret technical research findings owing to differences in language, terminology and conceptual frameworks [26, 36]. Bureaucratic hurdles and rigid institutional structures also obstructed collaboration, as seen in studies where lengthy approval processes and complex governance frameworks delayed stakeholder engagement [30, 31, 49].

Another major barrier was cultural and structural resistance to integrating research into policy, particularly in regions where evidence-informed policymaking was not an established norm [22, 39, 48]. Institutional inertia, scepticism about external research, and reluctance to change existing policies limited the uptake of research findings. Some studies also highlighted challenges related to stakeholder turnover, where changes in leadership or government administration disrupted ongoing collaborations, necessitating renewed engagement efforts [16, 21, 41].

### Facilitators for effective collaboration

Despite these challenges, several facilitators were identified that enhanced collaboration between researchers and policymakers. Structured governance and leadership played a crucial role in ensuring that research efforts aligned with policy priorities [9, 30, 31, 41]. Establishing governance bodies such as policy advisory groups and dedicated research-policy liaison offices created sustained engagement mechanisms, improving the application of research evidence.

Early and sustained stakeholder engagement was another critical factor. Studies found that involving policymakers from the research design phase ensured that studies were relevant, feasible and aligned with policy priorities [22, 48, 51]. The use of formalized engagement mechanisms, such as co-production workshops and iKT frameworks, significantly improved collaboration outcomes [9, 36, 39].

The presence of iterative feedback mechanisms was also beneficial in maintaining engagement between researchers and policymakers. Digital platforms, structured feedback loops and regular policy briefings were effective tools in bridging communication gaps and ensuring that research findings remained adaptable to evolving policy needs [26, 30, 31]. Studies further emphasized that capacity-building initiatives enhanced policymakers’ ability to interpret and apply research findings effectively [13, 16, 28]. Training programs, knowledge-sharing workshops and cross-sector mentorship programs facilitated the development of research literacy among policymakers, improving the uptake of research evidence in decision-making.

Finally, embedding research within policymaking structures was highlighted as a key enabler. Programs such as the Thanzi Programme established dedicated Health Economics and Policy Units (HEPUs), which served as intermediaries between researchers and government bodies, ensuring continuous knowledge exchange [28]. Similarly, integrated knowledge translation networks in several studies fostered sustained collaboration by institutionalizing researcher-policymaker interactions [14, 26].

Table 1 summarizes the main barriers and facilitators to research–policy engagement identified in the scoping review, organized by thematic domains. While not exhaustive, the table illustrates how specific facilitators have been used to address commonly reported challenges across diverse health system contexts.

### Outcomes of effective collaboration

Effective collaboration between researchers and policymakers led to several tangible outcomes. Increased research relevance and applicability was a common finding, with co-produced research demonstrating higher alignment with real-world policy challenges. Studies such as Williamson et al. [51] and Jessani et al. [16] found that sustained partnerships ensured that research outputs were tailored to meet specific policy needs, increasing their utility and uptake.

Another key outcome was the enhanced implementation of research findings. Studies reported that active stakeholder engagement and continuous communication facilitated the direct application of research recommendations into policy reforms [9, 36, 43]. In some cases, the adoption of co-produced research led to legislative changes and new funding allocations for public health programs [13, 27, 30, 31].

Strengthened networks and relationships also emerged as a major benefit of collaborative research efforts. Studies found that long-term partnerships between researchers and policymakers helped build trust, mutual understanding, and institutional memory, reducing future barriers to research-policy integration [16, 36, 46]. The development of communities of practice and policy-research networks played a significant role in sustaining these relationships over time [21, 22].

Furthermore, several studies highlighted that effective collaboration resulted in the joint development of policy tools, such as evidence briefs, policy guidelines and decision-support frameworks. Kalibala and Nutley [17] and Leslie et al. [22] documented how structured stakeholder engagement led to the co-design of implementation strategies tailored to local contexts. These collaborative efforts ensured that research findings were not only disseminated but also operationalized within policy frameworks.

Another significant outcome was capacity building and skill development among policymakers and researchers. Training programs, structured dialogues and interactive workshops contributed to enhanced research translation skills, ultimately improving decision-making processes [10, 13] (Chung et al. 7). These capacity-building efforts facilitated a more evidence-informed policymaking culture, particularly in settings where research utilization had previously been limited [28, 39].

Lastly, improved public health interventions were reported as a direct result of effective researcher-policymaker collaboration. Studies demonstrated that research-driven policy adaptations led to improved health service delivery, resource allocation and public health outcomes [14, 24, 30, 31]. Examples included policy changes targeting non-communicable diseases, enhanced mental health service frameworks and strengthened environmental health policies in response to climate change challenges.

These findings collectively underscore the importance of structured collaboration strategies in ensuring that research meaningfully contributes to policy and practice, ultimately improving health outcomes and public policy effectiveness.

**Fig. 2:**
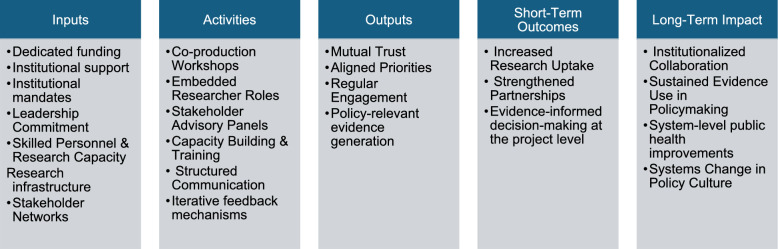
Logic model for enhancing research–policy collaboration in public health

Figure 2 presents a logic model developed from the findings of this scoping review to illustrate how strategic collaboration between researchers and policymakers can be structured to drive systemic change in public health. The model traces a pathway beginning with essential inputs such as dedicated funding, institutional support, leadership commitment, research capacity, infrastructure and stakeholder networks, which create the conditions for collaborative action. These inputs support a range of targeted activities including co-production workshops, embedded researcher roles, stakeholder advisory panels, capacity building and training initiatives, structured communication and iterative feedback mechanisms. Together, these activities produce immediate outputs such as mutual trust, aligned priorities, regular engagement and the generation of policy-relevant evidence. These outputs lead to short-term outcomes including increased research uptake, strengthened partnerships and evidence-informed decision-making at the project level. Over time, these developments contribute to long-term impacts such as institutionalized collaboration, sustained use of evidence in policymaking, system-level public health improvements and critically, a shift in policy culture. In this new culture, EIDM becomes embedded as a normative practice. This model supports the central aim of the review: not only to identify effective strategies, but to illuminate how they can be embedded, scaled and institutionalized across diverse policy contexts to produce lasting impact.

### Cross-contextual insights and comparative patterns

Comparative patterns across regions and themes reveal that collaboration strategies are highly contingent on local institutional environments and sectoral demands. In LMICs such as Kenya, Nigeria and Uganda, collaborative engagement frequently relied on external support mechanisms such as donor-funded initiatives, embedded researchers and technical advisors. These approaches, as exemplified in the Thanzi Programme [28], prioritized capacity building and relational trust to address weak or fragmented formal structures. Similarly, iKT strategies in sub-Saharan African countries often focused on tailored engagement with ministries and NGOs, aiming to build research-use capabilities and foster inclusive networks [27, 39].

In contrast, high-income countries (HICs) such as Canada, the UK and Australia employed more institutionalized collaboration mechanisms, including knowledge translation platforms, structured policy fellowships and integrated governance models [9, 14, 40]. These mechanisms were embedded within formalized systems that supported sustained interaction and evidence integration into policy processes.

Thematic variation was also evident: trust-intensive domains such as mental health and Indigenous health emphasized sustained engagement and co-design, as shown in Canadian studies addressing deinstitutionalized mental healthcare [10] and Indigenous food systems [48]. In contrast, broader health system and non-communicable disease (NCD) collaborations leaned toward structured, indicator-driven policy platforms, especially in multisite implementation frameworks across sub-Saharan Africa [27, 39]. These contrasts underline the importance of adapting collaboration strategies to fit local political, institutional and thematic contexts addressing a key gap in comparative insight across regions and sectors.

## Discussion

The findings of this scoping review align closely with the “Embedding Research in Health Policy and Systems” (ERHPS) approach, which emphasizes the institutional integration of researchers into policymaking processes to support real-time evidence use, enhance policy relevance and promote long-term sustainability. As articulated by the World Health Organization’s Alliance for Health Policy and Systems Research [42], the ERHPS model promotes key principles such as mutual priority settings, embedded researcher roles, iterative engagement and institutional co-governance, all of which were reflected in the four themes identified in this review.

Central to these themes is the concept of co-production, which underpins the ERHPS model’s emphasis on shared problem definition, mutual accountability and continuous researcher–decision-maker collaboration. Rather than treating co-production as a participatory add-on, ERHPS positions it as a structural condition that enhances both the contextual fit and policy relevance of evidence [42]. This framing is supported by empirical initiatives such as the Australian Prevention Partnership Centre [13] and the Thanzi Programme [28], where researchers worked in embedded, hybrid roles to support real-time policy development.

A key insight from this review is the dual character of embedding strategies. Co-production can manifest either as a deliberate intervention or as an emergent outcome. In the first mode, embedding was pursued through structured mechanisms that institutionalize researcher–policy collaboration. These included policy fellowships [13], embedded research units [28] and co-governance platforms [31, 40, 51]. Additional examples included formalized co-creation models and structured advisory roles [14, 17], as well as joint institutional arrangements for evaluation and learning [23, 26]. These mechanisms were intentionally designed to foster continuous engagement, shared authority and accountability between researchers and decision-makers.

In contrast, the second mode describes co-production as a relational outcome that develops organically through repeated interactions, trust-building and shared experience over time. This distinction reflects an evolving understanding of embedded research and resonates with previous studies, such as Oliver et al. [33] and Kneale et al. [18], which emphasize that while structural mechanisms are important, relational dynamics including mutual respect and informal engagement often determine whether evidence is actually used.

Our findings illustrate this duality across diverse contexts. In some studies (e.g., [13, 36]), co-production was institutionalized through intentional design and role definition, producing faster, more visible results and enabling easier monitoring and scaling. In others (e.g., [9, 28]), it emerged from long-term engagement and relational trust, offering greater resilience but proving harder to replicate. Both approaches contributed meaningfully to research–policy integration, although they differed in replicability, durability and dependency on institutional or relational capital. Intervention-based co-production allowed for formal accountability and clearer uptake pathways, while outcome-based co-production offered adaptive advantages in politically dynamic or resource-constrained settings. This layered insight reinforces the need, identified by Gagliardi et al. [11] and Sajadi et al. [38], to balance structured embedding with flexible, trust-based collaboration tailored to local institutional conditions.

These findings are consistent with the conceptual foundations articulated by WHO [42], which argues that embedding research requires attention to both institutional design and relational engagement. Similar to the intervention–outcome distinction drawn here, Oliver et al. [33] highlight that evidence use in policymaking depends as much on informal relationships and embeddedness as it does on formal channels. Gagliardi et al. [11] and A. Koon and Rao [20] likewise caution that technical solutions such as knowledge brokers or research platforms may fall short without the cultivation of mutual trust and iterative engagement. Our synthesis adds empirical clarity by categorizing co-production modes and showing how hybrid arrangements that combine formal structures with ongoing relationships can strengthen the resilience and relevance of evidence use in diverse health system contexts.

Capacity building in this context goes beyond technical training to encompass mutual learning and institutional capacity to demand, interpret and act on evidence. ERHPS views capacity as a systemic function, where the absorptive capacity of the system is built through embedded relationships, mentorship, and iterative learning. For instance, Mpando et al. [27] describe how Malawi’s Ministry of Health integrated research training within its policy cycles, while Jessani et al. [16] emphasize the value of embedded fellowships in Kenya. These findings align with Onwujekwe et al. [34], who emphasize the need for reciprocal capacity strengthening between researchers and policymakers as a foundation for long-term collaboration. This mirrors earlier insights from Bennett et al. [3], who argued that sustainable capacity building must be embedded within the organizational culture of health systems, rather than delivered through isolated workshops or external training modules. In addition, Newman et al. [29] observed that relational embeddedness and long-term mentorship models were key to increasing policymakers’ ability to engage with evidence in decision-making. Such comparative literature supports the ERHPS emphasis on capacity as a co-produced and embedded function, rather than a technical or transactional activity.

Structured communication mechanisms, such as embedded feedback loops, joint planning platforms and policy dialogues were also core to the ERHPS paradigm. Communication in this context is not a supplementary activity but an integrated function of embedded research partnerships. For example, Pearce et al. [36] describe the establishment of a Health Policy Partnership Unit in the Pacific, which institutionalized communication through co-produced policy briefs and embedded learning loops with ministry stakeholders. Similarly, Nguyen et al. [31] report on national immunization platforms in Southeast Asia that formalized communication between researchers and decision-makers via structured planning dialogues and routine evidence synthesis cycles. These mechanisms fostered joint sense-making and timely policy response in complex health systems.

In global partnerships, Voller et al. [50] highlight how both formal and informal communication channels were essential for knowledge integration across institutional boundaries. These findings reinforce the conclusion that sustained dialogue and institutionalized communication pathways are foundational to effective embeddedness, especially in complex health systems.

Institutional governance also emerged as a critical scaffolding that sustains research–policy collaboration. Mechanisms such as steering committees, liaison officers and embedded research units, including the Health Economics Units in Uganda and Malawi, transformed knowledge production into a shared governance function. As argued by Koon et al. [19], embedding governance mechanisms operationalizes shared authority, allowing researchers and policymakers to co-steer health systems. This institutional anchoring reduces dependence on individuals and buffers against political or administrative turnover. Our findings align with the observations of Kneale et al. [18], who warn that embedded efforts lacking governance integration often fail to achieve system-level transformation. These insights also echo Gilson et al. [12], who emphasize that health systems governance must be informed by relational and contextual understanding, supporting co-produced knowledge and policy learning through interdisciplinary, embedded approaches.

The barriers identified in this review, including misaligned institutional incentives, time and resource constraints, and fragmented bureaucracies, are precisely the systemic limitations that the ERHPS approach seeks to address. These challenges reflect those documented by [38], who highlight institutional resistance, weak leadership support and poor coordination as key obstacles to embedding evidence-informed priority setting. In both low- and high-income country contexts, our findings suggest that relational trust, while valuable, is not sufficient on its own. Structural mechanisms are essential to stabilize partnerships and insulate them from political or administrative volatility. This observation builds on insights from Gagliardi et al. [11], who caution that informal collaboration often fails when not reinforced by formal agreements and institutional mandates.

Facilitating conditions such as sustained funding, leadership engagement and alignment of research agendas with policy priorities have been consistently recognized as critical enablers of embedded research partnerships. Rather than treating these factors as universally effective, our review underscores the importance of understanding their function within distinct modes of embedding. Specifically, we found that in intervention-based models, these enablers support formal accountability structures and visibility, whereas in emergent models, their role is more diffuse, often materializing through iterative relationship-building and institutional familiarity. This differentiated insight contributes to a more nuanced understanding of the enabling environment for embedded research. It aligns with Hoekstra et al. [15], who identified leadership commitment, resource availability and trust as core to partnership effectiveness, and with Kneale et al. [18], who call for context-sensitive frameworks that can accommodate both structured and organically evolving research-policy arrangements.

The outcomes reported across the included studies, including improved policy responsiveness, enhanced mutual trust and greater research relevance, are broadly consistent with findings from earlier syntheses [11, 50]. However, this review adds value by showing that such outcomes are more likely to be sustained when embedding is conceptualized as a system-level reform rather than a discrete, time-bound project. In doing so, our synthesis bridges conceptual frameworks like ERHPS with the practical realities of policymaking in dynamic public health systems.

The logic model developed as part of this review further illustrates how embedding operates as a system-level process, linking inputs such as funding and personnel to activities such as co-production and communication, and to outputs including trust, engagement, and relevant evidence. These outputs then contribute to short-term outcomes such as increased research uptake and improved decision-making, and ultimately toward long-term impacts such as institutionalized collaboration and systems change in policy culture. By structuring these relationships, the model visually complements the ERHPS framework, clarifying how both relational and structural dimensions interact to enable sustained evidence use. It also offers a potentially transferable tool for policymakers and researchers seeking to design, evaluate or adapt embedded research strategies across diverse health system contexts, while remaining sensitive to local structures and governance realities.

This review has several strengths. It employed a systematic and comprehensive approach to identify and synthesize diverse forms of collaboration between researchers and policymakers. The use of an established scoping review methodology allowed for the inclusion of a broad range of study designs and evidence types, providing a wide-angle view of collaborative dynamics across settings. Importantly, this review contributes conceptual clarity by identifying two distinct modes of co-production: one as a deliberate intervention, the other as an emergent outcome, highlighting how collaboration unfolds differently across policy contexts.

These findings have important implications for practice. To move beyond short-term or project-based collaboration, public health agencies, academic institutions and funding bodies should prioritize the institutionalization of research–policy partnerships. First, embedding co-production mechanisms such as dedicated liaison offices, policy fellowships or embedded research units can ensure sustained engagement between researchers and policymakers. Second, mutual capacity-building efforts are essential: policymakers should receive structured training in research literacy and critical appraisal, while researchers must be equipped with skills in policy communication and stakeholder engagement. Third, establishing iterative communication mechanisms such as policy roundtables, regular evidence briefings and feedback loops can enhance the relevance and timeliness of research for policy needs. Finally, funding frameworks should incentivize and, where appropriate, require demonstrable collaboration structures and shared ownership of outputs. Taken together, these strategies can help institutionalize the integration of evidence into policy, making it a systematic feature of public health governance rather than an exception.

## Conclusions

This review demonstrates that embedding research into health policy systems is not a one-off intervention, but a structural transformation. Co-production, capacity building, institutionalized communication and governance are not separate strategies; they function as interconnected pillars within embedded research ecosystems. Together, they help shift evidence use from sporadic translation to a routine element of public health governance.

By distinguishing between embedding as a deliberate intervention and as an emergent outcome, this review deepens the ERHPS model and highlights the practical value of hybrid approaches. These findings offer a usable framework for institutions aiming to formalize research–policy collaboration, particularly in dynamic or resource-limited environments.

What is needed now is not more short-term experiments, but long-term investment in shared authority, continuous engagement and adaptive infrastructure. Embedding should be understood and implemented as a system reform strategy that reconfigures how knowledge is produced, interpreted and applied across public health institutions.

Future research should evaluate the effectiveness of different embedding models, develop robust frameworks to measure their impact and explore how these approaches can be adapted across diverse political and institutional contexts. Longitudinal studies are especially important to assess sustainability over time, alongside deeper analysis of resource requirements, equity dynamics and power relationships within embedded research partnerships.

## Supplementary Information

Additional file1 (DOCX 15 kb)

Additional file2 (DOCX 94 kb)

## Data Availability

No datasets were generated or analysed during the current study.
