# Supplementary material for: Facilitating collaboration between public health researchers and policymakers: a scoping review of global practices, barriers and facilitators
Source: Health Res Policy Syst. 2026 Jan 21;24:17. doi: 10.1186/s12961-026-01443-y (PMC12908259; doi:10.1186/s12961-026-01443-y)
Supplement: Supplementary file 1 — Additional file1 (DOCX 15 kb) [file 12961_2026_1443_MOESM1_ESM.docx]

Appendix 1: Search strategies for each databases

| Database | Search terms |
| --- | --- |
| Medline (via PubMed) | ("knowledge translation" OR "Knowledge Transfer" OR "Knowledge Exchange" OR "Knowledge Utilization" OR "Knowledge Mobilization" OR " Knowledge Integration" OR " Knowledge Dissemination" OR" Knowledge Implementation" OR "Research Translation" OR " Research Utilization "OR" Knowledge Application" OR "Information Translation" OR "Translational Science" OR "Research-to-Practice" OR "Knowledge Conversion" OR "Knowledge Transfer and Exchange" OR "Research Communication" OR "Integrated Knowledge translation" OR " Integrated knowledge transfer" OR "partnership research" OR "Participatory approach" OR "Collaborative research" OR "Co-production of Knowledge")AND (Policymakers OR" Decision makers" OR Decision-makers OR Stakeholders OR "Government officials" )AND (Researchers OR Academics OR Academia OR Scholars OR Scientists )AND (strategy or strategies OR practice OR model)AND(“Public health”) |
| SCOPUS | TITLE-ABS-KEY("Knowledge Translation" OR "Knowledge Transfer" OR "Knowledge Exchange" OR "Knowledge Utilization" OR "Knowledge Mobilization" OR "Knowledge Integration" OR "Knowledge Dissemination" OR "Knowledge Implementation" OR "Research Translation" OR "Research Utilization" OR "Knowledge Application" OR "Information Translation" OR "Translational Science" OR "Research-to-Practice" OR "Knowledge Conversion" OR "Knowledge Transfer and Exchange" OR "Communication" OR "Integrated Knowledge translation" OR "Integrated Knowledge Transfer" OR "partnership research" OR "Participatory Approach" OR "Collaborative Research" OR "Co-Production of Knowledge") AND TITLE-ABS-KEY("Policymakers" OR "Decision Makers" OR "Decision-Makers" OR "Stakeholders" OR "Government officials") AND TITLE-ABS-KEY("Researchers" OR "Academics" OR "Academia" OR "Scholar" OR "Scientists") AND TITLE-ABS-KEY ("Strategy" OR "Strategies" OR "Practice" OR "Model") AND TITLE-ABS-KEY("Public Health"). |
| Web of Science | ("knowledge translation" OR "Knowledge Transfer" OR "Knowledge Exchange" OR "Knowledge Utilization" OR "Knowledge Mobilization" OR " Knowledge Integration" OR " Knowledge Dissemination" OR" Knowledge Implementation" OR "Research Translation" OR " Research Utilization "OR" Knowledge Application" OR "Information Translation" OR "Translational Science" OR "Research-to-Practice" OR "Knowledge Conversion" OR "Knowledge Transfer and Exchange" OR "Research Communication" OR "Integrated Knowledge translation" OR " Integrated knowledge transfer" OR "partnership research" OR "Participatory approach" OR "Collaborative research" OR "Co-production of Knowledge")AND (Policymakers OR" Decision makers" OR Decision-makers OR Stakeholders OR "Government officials" )AND (Researchers OR Academics OR Academia OR Scholars OR Scientists )AND (strategy or strategies OR practice OR model)AND(“Public health”) |
| Embase-213 | ('knowledge translation'/exp OR 'knowledge translation' OR 'knowledge transfer'/exp OR 'knowledge transfer' OR 'knowledge exchange'/exp OR 'knowledge exchange' OR 'knowledge utilization' OR 'knowledge mobilization' OR 'knowledge integration' OR 'knowledge dissemination' OR 'knowledge implementation' OR 'research translation' OR 'research utilization' OR 'knowledge application' OR 'information translation' OR 'translational science'/exp OR 'translational science' OR 'research-to-practice' OR 'knowledge conversion' OR 'knowledge transfer and exchange' OR 'research communication' OR 'integrated knowledge translation'/exp OR 'integrated knowledge translation' OR 'integrated knowledge transfer' OR 'partnership research' OR 'participatory approach' OR 'collaborative research'/exp OR 'collaborative research' OR 'co-production of knowledge') AND ('policymakers'/exp OR policymakers OR 'decision makers' OR 'decision-makers' OR stakeholders OR 'government officials'/exp OR 'government officials') AND (researchers OR academics OR 'academia'/exp OR academia OR scholars OR 'scientists'/exp OR scientists) AND ('strategy'/exp OR strategy OR strategies OR 'practice'/exp OR practice OR 'model'/exp OR model) AND ('public health'/exp OR 'public health') |
