# Supplementary material for: Facilitating collaboration between public health researchers and policymakers: a scoping review of global practices, barriers and facilitators
Source: Health Res Policy Syst. 2026 Jan 21;24:17. doi: 10.1186/s12961-026-01443-y (PMC12908259; doi:10.1186/s12961-026-01443-y)
Supplement: Supplementary file 2 — Additional file2 (DOCX 94 kb) [file 12961_2026_1443_MOESM2_ESM.docx]

**Appendix 2: Detailed data extraction information from the 24 studies included in the review**

| **Title** | **Author(s)** | **Study population** | **Research design** | **Objective**  **+ strategy or intervention** | **Outcome measures** |
| --- | --- | --- | --- | --- | --- |
| 1. Research-to-policy partnerships for evidence-informed resource allocation in health systems in africa: an example using the thanzi programme | Nabyonga et al.,  2024  Africa | The participants in the thanzi programme include a broad range of stakeholders such as in-country researchers, health policymakers, and academic and research institutions.  (the number is not specified). | They did not mention a research design. They only describe the use of framework approach (.  This framework comprises three interrelated pillars: research evidence generation, capacity and capability building, and research-to-policy engagement. Each of these pillars involves a range of activities aimed at integrating research into policy-making processes effectively. | Objective: to explore the application of a research-to-policy partnership framework used by the Thanzi Programme. This framework is aimed at supporting evidence-informed health resource allocation decisions in Africa. It combines high-quality multidisciplinary research, sustained engagement between researchers and policymakers, and capacity building to effectively guide and support policymakers​​.  Strategy:   - Interdisciplinary Research and Evidence Generation: The program conducted robust, locally relevant research to generate evidence that could guide policy and decision-making processes. This involved health economics, epidemiological modeling, and political science, focusing on aligning the research with real-world policy priorities. - Capacity and Capability Building: There was a strong emphasis on strengthening the institutional and individual capacity to use research evidence. This included training programs, workshops, and creating platforms for continuous learning and engagement between researchers and policymakers. - Structured Policy Engagement: The program facilitated structured engagement through various platforms to ensure ongoing dialogue between researchers and policymakers. This included establishing Health Economics and Policy Units (HEPUs) which served as intermediaries between ministries of health and academic institutions to promote evidence-based policymaking. - Community of Practice: At a regional level, a Health Economics Community of Practice was established to foster dialogue, share best practices, and enhance the use of health economics evidence in policy processes across the member states.   These strategies underscored a multi-layered approach to integrating research with policy by focusing not only on evidence generation but also on building the capacity to use this evidence and establishing continuous, structured engagement between all stakeholders involved | Barriers   - There is often a limited amount of high-quality, locally relevant research available. This lack can prevent policymakers from having timely access to data that directly addresses the health issues pertinent to their specific contexts. - The ability to implement recommendations from research is frequently hindered by the slow production of evidence and the lack of comprehensive data that encompasses all necessary aspects of policy implementation. - There is a notable deficiency in local expertise to critically evaluate and interpret research outputs. This includes understanding economic evaluations and their implications, which are crucial for informed decision-making. - Financial limitations and local political circumstances often restrict the feasibility of implementing research recommendations. Budgetary processes may not align with the timing or requirements of integrating new research findings, and political volatility can undermine sustained support. - Poor communication strategies and ineffective dissemination of research findings to policymakers can lead to underutilization of valuable insights. Additionally, the absence of interactive approaches to make evidence locally relevant and updated can further limit its impact.   Facilitators   - Establishing formal structures such as Health Economics and Policy Units (HEPUs) and regional communities of practice provides continuous and structured opportunities for interaction between researchers and policymakers. These platforms help in shaping research agendas and ensuring that evidence is both generated and shared in ways that align with policy needs. - Involving policymakers in the early stages of research design helps ensure that the studies are relevant and directly applicable to current policy challenges. This collaborative approach increases the likelihood that the research outputs will be used in decision-making. - Developing training programs and curricula in health economics and policy-related topics addresses the critical skills gap among local researchers and policymakers. This not only improves the quality of local research but also enhances the ability of policymakers to understand and apply research findings. - Policy units like HEPUs serve as bridges between academia and government, facilitating the direct transfer of research insights into the policy-making process. Similarly, practice communities bring together experts to focus on specific issues, promoting the use of economic evidence in regional policy processes. |
| 1. Producing knowledge together:   A participatory approach to synthesising  Research across a large‑scale collaboration  In Aboriginal and Torres Strait Islander health | Conte et al., 2024  Australia | The participants included twelve researchers and eleven service providers or policy partners, of whom eight self-identified as Aboriginal and/or Torres Strait Islander | They did not mention a specific study design.  The study design was centered around a participatory approach to synthesizing evidence across a large-scale collaboration, specifically focused on Aboriginal and Torres Strait Islander health | Objective: to describe a process an innovative participatory process of synthesizing a large body of academic research and compare the findings from this participatory synthesis against two traditional approaches: a rapid review and a structured review. This approach is aimed at strengthening primary health care for Aboriginal and Torres Strait Islander peoples by involving diverse stakeholders in the synthesis of evidence, enhancing the relevance and utility of the synthesized knowledge​​.  Strategy: they use (Participatory Workshops)  Two facilitated workshops totalling of four hours were conducted, engaging stakeholders in an active review and discussion of the synthesized evidence. These workshops were designed to leverage the experiential knowledge of the participants, particularly the Aboriginal and Torres Strait Islander service providers and policy partners, to enrich the academic research findings with contextual insights.  This participatory synthesis was not a one-off event but an iterative process involving continuous participant feedback, ensuring comprehensive findings aligned with end-users' needs and priorities, enhancing the overall understanding of the subject matter. | Barriers:   - Specialized skills and training required for effective participation in evidence synthesis, which can be a hurdle due to the technical nature of the processes involved. - Time constraints and the extensive commitment needed from participants to engage deeply with the synthesis processes​​.   Facilitators:   - Engaging stakeholders as equal partners in the knowledge co-production process helps in making the synthesized evidence more applicable and actionable. - Use of structured facilitation during participatory workshops aids in effectively managing the synthesis process and ensuring that diverse perspectives are incorporated into the synthesis outcomes   Outcome:  The participatory synthesis was found to generate the most detailed and unique findings, providing contextual insights about the relevance of key messages for practice. This method was compared to traditional synthesis methods, with the participatory approach showing advantages in extending knowledge and identifying lessons most relevant to the participants, who are the end-users of the research​​. |
| 1. Developing, implementing, and monitoring tailored strategies for integrated knowledge translation in five sub-Saharan African countries | Sell et al.,  2023  Sub-Saharan African | Participants included researchers and policy and practice partners across the five countries (Ethiopia, Malawi, Rwanda, South Africa, and Uganda)  (33 interviews with researchers and stakeholders from policy and practice,  And 31 survey responses), 49 documents, and eight responses to the reflective survey informed this article | A mixed-method IKT evaluation | Objective: to explore the development, implementation, and monitoring of IKT strategies in Ethiopia, Malawi, Rwanda, South Africa, and Uganda. It aimed to enhance the relevance and use of research in public health decision-making through tailored IKT strategies.  Strategies: five tailored/site-specific IKT strategies were implemented in the five countries focusing on engaging with stakeholders to enhance the use and uptake of high-quality, contextualized evidence into public health and healthcare decision-making. Each country's strategy is customized based on its local context, stakeholders, and priorities, aiming to establish partnerships, share research updates, collaborate on capacity-building activities, and bridge the evidence-policy-practice gap for non-communicable diseases and road traffic injuries prevention and care.   1. Ethiopia   Focus: Strengthening and establishing partnerships with policymakers at the Ethiopian Ministry of Health, NGOs active in NCD healthcare, advocacy organizations, and other academic institutions.  Goals:  Sharing research updates, collaborating on capacity-building for Evidence-Informed Decision Making (EIDM), and establishing a national NCD research network to integrate efforts across various stakeholders for NCD prevention and control.   1. Malawi   Focus: Engaging with the Ministry of Health’s NCD department and the ministry’s Knowledge Translation Platform (KTP).  Goals:  To bolster evidence synthesis, capacity-building, and the dissemination of research results within the realm of NCD care.   1. Rwanda   Focus: Prioritized engagement with the Ministry of Health, Rwanda Biomedical Center, Rwanda Utility Regulatory Authority, Rwanda Transport Development Agency, Ministry of Public Service and Labor, Ministry of Sports, and the Traffic Police Department.  Goals:  Stakeholder meetings, training for data collectors, manuscript development, capacity-building, issue brief development, and dissemination efforts.   1. South Africa   Focus: Increasing the uptake of research evidence in policy and practice.  Goals:  Prioritized engagement with NCD directors in national and provincial health departments.  Activities included stakeholder meetings, an NCD symposium, policy dialogues, conference presentations, peer-reviewed publications, webinars, issue briefs, emails, and in-person meetings.   1. Uganda   Focus: Building upon existing road safety work  Goals:  The intervention aims to formalize stakeholder engagements to address staff turnover and enhance road safety initiatives. The approach includes formal communications, phone calls, physical visits, and in-person meetings for planning and dissemination purposes. | Facilitators and Barriers  Capacity Building and Skills: A primary facilitator is the emphasis on capacity-building to ensure sufficient skills in IKT and Evidence-Informed Decision Making (EIDM). The document points out the need for broad technical and interpersonal skills for effective IKT, noting that current education and training programs for health professionals rarely include such training. CEBHA+ aimed to address this by developing and implementing a course on evidence-based public health and integrating it into institutional curricula​​.  Outcomes  Continuous Stakeholder Engagement: Adherence and reliability to continuous stakeholder engagement were high across the five sites, demonstrating the effectiveness of stakeholder involvement in the research process​​.  Tailoring of IKT Strategies: The strategies were tailored to individual stakeholder needs and context, which subsequently adapted as projects progressed. This flexibility allowed for adjustments based on stakeholder preferences, project-related tailoring due to delays in research, and adaptations to the macro context like parliamentary elections and the COVID-19 pandemic​​. |
| 1. Understanding how and under what circumstances integrated knowledge translation works for people engaged in collaborative research: metasynthesis of IKTRN casebooks | Dunn et al.,  2023  Canada | The participants include researchers, patients/public, clinicians, managers, and policymakers who are engaged in the collaborative research projects described in the Integrated Knowledge Translation Research Network (IKTRN) casebooks.  These stakeholders participated in various capacities, contributing to the identification of relevant problems, engaging in the conduct of research, and having the authority or ability to implement research recommendations​​. | This study used metasynthesis approach using a qualitative research  Design, to examine IKT case studies.  (Qualitative study) | Objective: to examine how research co-production works through a metasynthesis of IKTRN casebooks. It aimed to extend existing theoretical and empirical perspectives about the co-production process in collaborative research, focusing on the interaction and collaboration between diverse stakeholders​​.   - This study focused on 4 research questions:  1. What contextual factors and processes facilitated use of an IKT approach for research co-production? 2. What were the barriers and challenges to using an IKT approach for research co-production? 3. What IKT activities were used to increase the likelihood of successful research co-production? 4. What outputs and outcomes resulted from using an IKT approach to research co-production?   • This study used a theoretical framework to guide its metasynthesis of IKT casebooks. Specifically, the study employed a modified realist review approach as the underlying theoretical framework  Strategies:   - Stakeholder Engagement: Engaging a broad group of stakeholders—including clinicians, policymakers, patients, and managers—is emphasized. This engagement involves involving the right people at the right time and in the right way, to ensure that all perspectives are considered in the research process. - Partnership Building: The creation and maintenance of partnerships are central to the collaboration strategy. This includes co-investigation where knowledge-users and researchers work together from the formulation of research questions to the dissemination of findings. - Communication Strategies: Regular and effective communication is a cornerstone of successful collaboration. This may involve using various platforms such as newsletters, email updates, and regular meetings to ensure all partners are kept informed and engaged. - Joint Decision-Making and Governance: Establishing a joint governance structure for the research project, which includes shared decision-making processes, ensuring that all parties have a say in key decisions. - Capacity Building and Training: Providing training and support to all partners to ensure they have the necessary skills and understanding to participate fully in the research process. - Feedback Mechanisms: Using iterative feedback loops to refine and adjust the research process based on the input from all stakeholders, ensuring that the research remains relevant and aligned with the needs of the users. - Use of Digital Tools and Platforms: Although not explicitly detailed in the snippets, collaboration in modern research settings often utilizes digital platforms for document sharing, data collection, and communication to facilitate the involvement of diverse stakeholders who might not be co-located.   These methods are structured to address common barriers such as competing priorities, differing organizational cultures, and the complexity of integrating different types of expertise. By systematically applying these strategies, the study aims to foster a productive and effective collaborative environment for IKT. | Outcomes  1. What contextual factors and processes facilitated use of an IKT approach for research co-production?  Outcome:  The study identified several contextual factors and processes that facilitated the use of an IKT approach. These included strong leadership, organizational support, alignment of the research with the interests and priorities of the stakeholders, and the presence of dedicated resources such as time and funding. Additionally, the establishment of clear communication channels and structured engagement processes helped in facilitating collaboration between researchers and knowledge users.  2. What were the barriers and challenges to using an IKT approach for research co-production?  Outcome: Numerous barriers and challenges were highlighted, including:   - Resistance from traditional research environments which may not fully support participatory approaches. - Limited funding and resources, which can restrict the scope of activities and the sustainability of partnerships. - Differences in language and goals between researchers and practitioners or policymakers, making it difficult to align objectives and expectations.   Time constraints, as participatory approaches can be more time-consuming than traditional methods.  3. What IKT activities were used to increase the likelihood of successful research co-production?  Outcome: Several effective IKT activities were noted:   - Regular and structured meetings to facilitate ongoing dialogue and engagement. - Joint planning and decision-making, where researchers and knowledge users collaboratively developed research questions, methods, and dissemination strategies. - Training sessions for both researchers and stakeholders to understand each other’s fields better. - Use of communication tools like newsletters, web portals, or social media to maintain engagement and disseminate findings.   4. What outputs and outcomes resulted from using an IKT approach to research co-production?  Outcome: The use of IKT approaches led to several significant outputs and outcomes:   - Enhanced relevance and applicability of research findings to policy and practice. - Increased uptake and implementation of research outcomes due to the involvement of end-users in the research process. - Publications and policy documents that reflect the collaborative efforts of researchers and stakeholders. - Strengthened relationships and networks among diverse groups, which can facilitate future collaborations.   These outcomes demonstrate that despite challenges, employing an IKT approach can lead to meaningful engagement with stakeholders and the production of research that is more likely to be used and have a tangible impact on policy and practice. The insights from this metasynthesis help to further articulate how IKT can be effectively implemented and what factors contribute to its success, thereby supporting the advancement of research co-production methods.  Barriers   - Competing Priorities: Different organizations involved may have competing priorities that can impact their level of engagement and commitment to the project. - Complex Ethical and Organizational Processes: Navigating complex research ethics board approvals and organizational bureaucracies can delay or impede collaboration. - Resource Limitations: Constraints in funding, time, and personnel can restrict the ability of partners to engage fully in collaborative activities. - Communication Challenges: Inadequate communication can lead to misunderstandings and misalignments in goals and expectations among stakeholders. - Cultural and Structural Differences: Differences in organizational culture and structure between collaborating entities can lead to friction and inefficiencies. - Lack of Clear Governance Structures: Absence of clear governance and decision-making structures can lead to confusion and conflict among partners. - Resistance to Change: Organizational resistance to new processes and approaches introduced by the collaborative project can impede progress.   Facilitators   - Commitment to Collaboration: A shared commitment among all stakeholders to work together throughout the research process. - Support from Leadership: Strong backing from respected leaders within the collaborating organizations, which enhances buy-in and participation across levels. - Alignment with Organizational Priorities: Projects that are well-aligned with the interests, needs, and priorities of the participating sites and their stakeholders tend to receive more robust support and engagement. - Defined Roles and Responsibilities: Clear delineation of roles, responsibilities, and expectations helps in managing contributions and ensuring accountability among all partners. - Trust and Mutual Respect: Building an environment of trust and mutual respect among all partners, which facilitates open communication and sharing of information. - Effective Communication: Regular and clear communication strategies, including the use of digital tools and regular meetings, to keep all partners informed and engaged. - Flexibility and Adaptability: Allowing for flexibility in the collaboration process to accommodate changes and unexpected challenges. - Capacity Building: Investing in training and development to enhance the capabilities of all stakeholders to engage effectively in the research process. |
| 1. Combining public health evidence, policy experience and communications expertise to inform preventive health: reflections on a novel method of knowledge synthesis | Heenan et al., 2023  Australia | The participants included researchers, policymakers and communication experts involved in chronic disease prevention. The initiative was led by the Australian Prevention Partnership Centre, which collaborates extensively with Australia's National Health and Medical Research Council, various health departments, and nongovernment agencies. | They mention using Knowledge synthesis process | Objective: To reflect on a novel method of knowledge synthesis that integrates public health evidence with policy experience and communications expertise. This approach aims to support decision-makers in chronic disease prevention by making synthesized knowledge more accessible and actionable through co-production with policymakers.  Strategies:  The key components and process involved in this novel methos:   - Co-Production with Policymakers: The research method involves policymakers and stakeholders in the research process, ensuring the research questions and findings are relevant to policy needs and practical implementation. - Integration of Communications Expertise: Communications professionals are involved to ensure accurate and effective communication of findings. This involves developing various communicative outputs like reports, infographics, and podcasts that are tailored to the needs of the decision-makers and are easy to understand and use. - Systems Thinking: is applied to address complex interrelations within public health issues, illustrating how factors interact within the health system and influence outcomes. - Research-Policy Dialogues: are crucial for ongoing discussion and feedback, refining research focus, adapting findings to policy contexts, and discussing practical implications.   Application in Case Studies  The method was demonstrated through two case studies focusing on public health law and interventions in the first 2000 days of life. These case studies showed how the method could be applied to different public health topics to generate synthesized knowledge that is policy-relevant and readily applicable.  • The study indicated using theoretical frameworks in developing their novel method of knowledge synthesis. Specifically, the approach is underpinned by systems thinking and co-production. | Barriers:   - Complexity of public health issues which are often multi-faceted with various interdependent factors. - Challenges in integrating diverse types of evidence and experiences into a cohesive synthesis.   Facilitators:   - Co-production approach that directly involves policymakers and practitioners in the synthesis process, enhancing the relevance and applicability of the findings. - Strong support from a network of researchers and policy partners, which facilitates the integration of comprehensive expertise and perspectives.   Outcome  The main outcomes of the novel method are:   - Enhanced Policy Relevance: By integrating policy insights throughout the research process, the synthesized knowledge is highly relevant to policy-making. - Improved Accessibility of Findings: The use of strategic communications ensures that research findings are accessible and actionable for policymakers. - Increased Impact of Research: The collaborative approach helps bridge the gap between research and practice, potentially leading to more effective public health interventions   Overall, the main result is the establishment of a comprehensive and participatory method of knowledge synthesis that enhances the usability and impact of research findings in public health policy-making. |
| 1. A Research Translation, Implementation and Impact Strategy for the Australian Healthy Environments and Lives (HEAL)   Research Network | Lyne et al., 2023  Australia | Australian Healthy Environments and Lives (HEAL) Research Network involves a diverse group of participants including researchers from various academic institutes, Community members and organizations who actively involves in public health and environmental issues, Policymakers, practitioners, and service providers engaged in shaping and implementing health and environmental policies.  Aboriginal and Torres Strait Islander communities are central to the network, integrating their knowledge, wisdom, and experience in the research and decision-making processes. | The HEAL Network used a collaborative and integrative research design that emphasizes co-production, systems thinking, and multi-sectoral collaboration | Objective: to support improvements in health, the Australian health system, and the environment in response to the unfolding climate crisis. The network aims to protect and improve public health, reduce health inequities and inequalities, and strengthen health system sustainability and resilience in the face of environmental and climate change.  • The HEAL Network's strategy is informed by several theoretical framework: Health in All Policies (HiAP) and Environment in All Policies (EiAP), which ensure health and environmental considerations are integrated across all policy domains, and Research Translation theories, which focus on turning research findings into practical actions through knowledge mobilization and implementation science.  Strategies:  Key collaboration strategies of the Healthy Environments And Lives (HEAL) Network:   - Multi-sectoral Collaboration: Engages various sectors beyond health to address the climate crisis comprehensively. - Community and Stakeholder Engagement: Involves a broad range of stakeholders, including community members, researchers, policymakers, and practitioners to leverage diverse expertise. - Co-design with Communities: Prioritizes co-design methods with communities, especially integrating Aboriginal and Torres Strait Islander knowledge. - Knowledge Exchange and Translation: Facilitates the translation of research into policy and practice, ensuring research outcomes are accessible and applicable. - Capacity Building: Focuses on enhancing the capacity of stakeholders to participate effectively in research and policymaking. - Integrated Research Translation: Implements an integrated approach to research translation, engaging stakeholders throughout the research process. - Guiding Frameworks: Utilizes Health in All Policies (HiAP) and Environment in All Policies (EiAP) to integrate health and environmental considerations across policies.   These strategies aim to ensure that HEAL’s initiatives are inclusive, effective, and responsive to the needs of both the environment and public health. | Barriers:   - Cultural and institutional differences between researchers, communities, and policymakers, which can impede effective collaboration. - Limited existing relationships and engagement opportunities between stakeholders. - Conflicting priorities and resource constraints can affect the sustainability of initiatives.   Facilitators:   - Strong emphasis on co-production and community engagement, ensuring that research is relevant and tailored to the needs of diverse stakeholders. - Integrated approaches to knowledge exchange, which focus on mutual learning and sharing between researchers, policymakers, and community members. - Supportive frameworks and strategies that guide the HEAL Network's activities, such as the Health in All Policies (HiAP) and Environment in All Policies (EiAP) approaches.   Outcome:  The outcome of the HEAL Network's study is the development and implementation of a comprehensive strategy for research translation, implementation, and impact. This strategy effectively integrates scientific research into policy and practice to address public health challenges related to environmental and climate changes. Key achievements include establishing Communities of Practice for collaborative knowledge exchange, integrating Aboriginal and Torres Strait Islander Knowledge Systems into the research process, ensuring that Aboriginal and Torres Strait Islander perspectives are central to developing solutions for environmental and public health challenges, producing actionable policy tools such as policy briefs, and reports which are designed to assist policymakers and community leaders in making informed decisions , and enhancing capacity building across various stakeholder. Finally Implementation of a monitoring and evaluation framework that measures the effectiveness of research translation strategies and their impact on policy and practice. |
| 1. Enhancing the capacity of the mental health and substance use health workforce to meet population needs: insights from a facilitated virtual policy dialogue | Leslie et al., 2022  Canada | Participants included 46 stakeholders and policy decision-makers involved in the mental health and substance use health (MHSUH) workforce, representing a diverse array of sectors including healthcare providers, policy experts, and other relevant stakeholders from across Canada. | The study utilized a mixed-methods design that included a literature review, surveys of MHSUH providers, and key informant interviews. The final phase of the study was a 3-hour virtual policy dialogue designed to generate stakeholder-driven policy implications and priority actions based on the research findings. | Objective: to enhance the capacity of the MHSUH workforce in Canada, particularly in the context of the COVID-19 pandemic. The study aimed to identify key policy barriers impacting this essential workforce and to bridge the evidence-policy gap through a facilitated virtual policy dialogue.  Strategies:  They used virtual policy dialogue. | Barriers:   - The complexity of virtual settings and technology usage, which might limit participation from less tech-savvy individuals. - Time and resource constraints that could affect the ability of participants to engage fully in the dialogue process.   Facilitators:   - The use of technology-assisted facilitation, which enhanced equitable stakeholder participation and facilitated the management of discussions and consensus building. - Pre-circulated materials and structured dialogue sessions helped maintain focus and foster productive discussions.   Main outcome  The virtual policy dialogue not only generated rich qualitative insights that informed knowledge mobilization strategies and promoted evidence-informed policy but also highlighted several significant benefits of using virtual formats. Key outcomes from the dialogue included actionable policy recommendations, with equity serving as a foundational theme. The advantages of virtual formats enhanced stakeholder participation by providing increased accessibility, cost-effectiveness, and flexibility in scheduling, which facilitated deeper analysis and more efficient consensus building on critical policy priorities. These benefits ensured that the dialogue was inclusive and dynamic, allowing for comprehensive and informed decision-making that was aligned with the needs of the community. |
| 1. Annual gatherings as an integrated knowledge translation strategy to support local and traditional food systems within and across Indigenous community contexts: a qualitative study | Valaitis et al., 2022  Canada | The study involved 19 participants who are researchers, indigenous community members, local facilitators and partners from various sectors. | Qualitative study | Objective: The study aimed to support the development and implementation of Learning Circles in four Canadian Indigenous communities to improve local, community-based healthy food systems. It focused on using annual gatherings (AGs) as a platform for integrated knowledge translation (IKT) across diverse stakeholders, including researchers, Indigenous community members, and partners.  “The Learning Circles” refer to Local Healthy  Food to School initiative was to bring community members together with shared interest to develop a common vision to promote health and well being through the development of local, healthy food systems.  Strategies:  Annual Gatherings: This study demonstrates the effectiveness of annual gatherings as a collaborative strategy to facilitate knowledge translation and strengthen community-based food systems within Indigenous contexts. The emphasis on relationship building, trust, and aligning with Indigenous values was key to the project's success | Barriers:   - Aligning Research Agendas with Indigenous Cultural Values: Challenges included ensuring that diverse research agendas respect and integrate Indigenous cultural values, traditions, and priorities, which can significantly differ from conventional academic research frameworks. This alignment is crucial to ensure the relevance and acceptance of research outcomes within Indigenous communities. - Addressing Historical Tensions: Efforts were made to overcome past conflicts and residual mistrust between Indigenous communities and external researchers or institutions. These historical tensions arise from a long history of exploitation, misunderstanding, and disregard for Indigenous rights and perspectives in research and policy-making, necessitating a sensitive and informed approach to collaboration. - Overcoming Logistical Issues in Organizing In-Person Gatherings: Challenges involved managing the practical aspects of arranging face-to-face meetings, including booking suitable venues, coordinating travel for participants from diverse and often remote locations, and handling scheduling to accommodate various stakeholders. These logistical efforts are critical to ensuring broad and equitable participation, essential for the success of community-based participatory research.   Facilitators:   - Strong Community Leadership and Involvement: Leadership by community members drives project success, aligning activities with local values and enhancing engagement. - Prior Relationships and Trust-Building: Established relationships and ongoing engagement facilitate collaboration, especially important in communities with historical tensions. - Cultural Sensitivity and Respect: Respecting and integrating Indigenous cultural perspectives and knowledge systems into the research process bolsters participant involvement and enriches outcomes. - Technological Support for Virtual Participation: Technology enables virtual or hybrid participation, overcoming geographical and logistical barriers to maintain continuous engagement. - Flexible and Adaptive Meeting Structures: Adapting meeting formats to meet participant needs enhances involvement, accommodating diverse schedules and constraints. - Resource Allocation: Providing necessary resources, such as travel funding or compensation for time, ensures equitable participation and supports comprehensive stakeholder contribution. - Main outcome   The study, focusing on the use of Learning Circles within Indigenous communities to support local and traditional food systems, yielded significant results. It established that annual gatherings, as part of Learning Circles, effectively facilitated integrated knowledge translation among community members, researchers, and other stakeholders. These gatherings helped build relationships, align research agendas with Indigenous values, and enhance community engagement in food system initiatives. The results highlighted the importance of in-person, community-led discussions for building trust and enabling meaningful participation, which in turn supported actions at the local level to improve food systems. |
| 1. Value of consultation in establishing a public   Health research network: lessons from APPRISE | Smith et al., 2021  Australia | Participants included a wide range of stakeholders involved in infectious disease research and public health response: Researchers, Government representatives from health departments and agencies, Clinical professionals and medical emergency management personnel  Representatives from Aboriginal Community Controlled Health Organisations and other First Nations groups  Industry stakeholders from pathology laboratories, pharmaceutical, and technology companies | Two-stage modified Delphi process,  This process involved:  Stage one: Gathering input through one-on-one interviews and an online survey to identify and categorize feedback on the consultation process.  Stage two: Asking participants to rank the themes identified in the first stage regarding their influence or usefulness.  (mixed-methods) | Objective: to evaluate the impact of a consultative process on the establishment and early operation of APPRISE, a national research network for infectious disease preparedness. This included examining fulconsultation influenced research priorities, stakeholder engagement, and the integration of research into policy.  Strategies   - Broad Stakeholder Engagement: Engaging a wide array of stakeholders through interviews and workshops to ensure all voices were heard and integrated into the network's framework. - Use of Delphi Technique: Utilizing the Delphi method allowed for iterative feedback and consensus-building among a large group of experts without the need for physical meetings, accommodating the broad scope of the network   .  This evaluation provides valuable insights into the benefits and challenges of using extensive consultation to establish and manage a public health research network, emphasizing the importance of clear roles, managed expectations, and ongoing stakeholder engagement. | Barriers:   - Diverse Expectations: Balancing different stakeholder expectations, particularly between detailed research inquiries and the rapid response required by public health practices. - Logistical Challenges: Organizing effective consultation across a dispersed and diverse group of stakeholders.   Facilitators:   - Structured Consultation Process: A well-managed and phased consultation approach helped in systematically gathering and prioritizing stakeholder input. - Clear Communication and Commitment: Ongoing efforts to maintain clear communication lines and commitment from all parties involved were crucial for the success of the network.   Main Outcome:  The results highlighted several key outcomes from the consultation process:   - Consistent Research Priorities: Despite extensive consultations, the core research priorities remained unchanged, emphasizing the network's clear focus. - Strengthened Networking: The process significantly enhanced networking capabilities and stakeholder collaborations, particularly with government entities. - Engagement Challenges: Managing diverse and sometimes competing expectations between researchers and public health practitioners was a major challenge |
| 1. Integrated knowledge translation to strengthen public policy research: a case study from experimental research on income assistance receipt among people who use drugs | Mendell et al., 2021  Canada | 600 stakeholders, including policy makers, community organizations, and individuals with lived experience | A case study design | Objective: to focus on the impact of changing income assistance disbursement schedules among drug users in Vancouver, aiming to mitigate drug-related harms associated with synchronized monthly payments  Strategies: The IKT strategy, led by a knowledge broker, facilitated stakeholder engagement, development of knowledge translation products, and a multipronged approach to addressing barriers to evidence-informed public policy | Outcome:  This project shows how a multipronged approach to IKT addressed barriers to evidence-informed public policy and successfully contributed to increased public discourse around income assistance policy reform. Additionally, sustained engagement with diverse stakeholders led to improved contextual knowledge and understanding of potential community-level impacts that, along with scientific results, improved the evidence available to inform system change. This case study provides insight into the role IKT can play alongside research aimed at public policy improvements. |
| 1. Stakeholders’ experiences of the public health research process: time to change the system? | Laird et al., 2020  UK | The study population included 33 policy-makers, researchers,  Practitioners, funders, Knowledge brokers and directors of public  Health involved in generating or using research in  Outdoor space and non-communicable disease. (NCD) prevention in the United Kingdom  Were invited to take part via an email invitation. | Qualitative study | Objectives: to explore the perspectives and experiences of stakeholders involved in generating or using research in public health. This paper will specifically focus on outdoor space and NCD prevention to provide a focus for participants to discuss, but the findings are likely to be generalisable to other research areas in public health. The objectives are to understand (1) the challenges and enablers of stakeholder engagement in the  Research process and use of research evidence; (2) experiences of stakeholder engagement in the research process; and (3) the ways in which the research process could better facilitate stakeholder engagement and the use of research evidence.  Strategies:   - Regular Stakeholder Meetings: Regularly scheduled meetings helped maintain open lines of communication and allowed for ongoing adjustments to research processes in response to stakeholder feedback. - Stakeholder Workshops: Workshops and other interactive forums provided opportunities for stakeholders to directly engage with the research process, contributing their insights and perspectives in real-time. | Barriers:   - Structural and Systemic Issues: Participants noted significant systemic barriers within the public health research system, such as rigid funding structures that limit flexibility in research focus or methodology, and traditional metrics of success that may not fully capture the impact of public health initiatives. - Difficulties in Relationship Building:The challenge of establishing and maintaining productive and trusting relationships among diverse stakeholders (researchers, policymakers, community leaders) was frequently cited. Effective collaboration is often hindered by differing agendas, priorities, and timelines. - Translation of Research into Practice: There are considerable gaps in translating research findings into actionable policies or practices. This includes difficulties in communicating complex research outcomes to non-academic audiences and a lack of mechanisms to support the implementation of research into practice.   Facilitators  Effective Team Management: Positive experiences were associated with projects where there was clear, effective management of the research team, including structured communication strategies and defined roles and responsibilities.   - Cultural and Contextual Sensitivity: Research projects that were sensitive to the cultural and contextual nuances of the communities involved tended to be more successful. This includes designing research methodologies and outcomes that are relevant and appropriate to the local context. - Alignment with Stakeholder Needs: Projects that aligned closely with the needs and interests of the stakeholders involved facilitated greater engagement and were more likely to lead to successful outcomes. This alignment often required ongoing dialogue and adaptation of research goals.   Outcome:   - Stakeholders had positive experiences when engaged meaningfully in the research process, particularly when projects were well-planned and managed with attention to cultural and contextual factors. - The study highlighted several challenges, including structural and systemic issues, difficulties in building and maintaining relationships, and barriers in translating research into practice. - Suggestions included changing the funding system, exploring more collaborative research methodologies, and improving research translation and collaborative relationships throughout the research process. |
| 1. Knowledge translation for improving the care of deinstitutionalized people with sever mental health illness in health policy | Fulone et al., 2020  Brazil | The study participants involve Twenty-four individuals (policy-makers, stakeholders, researchers,  Representatives of the civil society, and public defense) | The study employed a multifaceted approach using the SUPPORT Tools for evidence-informed health policymaking. | Objective: to utilize knowledge translation (KT) tools to enhance the care of deinstitutionalized individuals with severe mental disorders in Brazil. The study focused on improving health policymaking through evidence-informed strategies to address the complex needs of this population.  The multifaceted approach that used the SUPPORT Tools for EIDM included:   - Capacity building - Identification of a priority policy issue - Meetings with policymakers, researchers, and stakeholders - Development of an evidence brief - Facilitating policy dialogue - Evaluation of the evidence brief and policy dialogue - Post-dialogue mini-interviews - Dissemination of findings   Strategies:   - Evidence Briefs and Policy Dialogues: These were used to consolidate research findings and facilitate comprehensive discussions among stakeholders. - Workshops: Conducted to raise awareness and train stakeholders on the use of SUPPORT tools and evidence-informed policymaking. - Regular Meetings: These were crucial for defining problems, developing solutions, and ensuring continuous engagement from all parties. | Barriers:   - Misalignments between research outcomes and policymaking needs. - Insufficient funding and resources to support comprehensive KT initiatives. - Cultural and systemic resistance to changes in mental health care approaches.   Facilitators:   - Strong engagement and collaboration among diverse stakeholders. - Use of structured KT tools like evidence briefs and policy dialogues to guide discussions. - Capacity building activities that enhanced stakeholders' understanding of evidence-informed policymaking.   Outcome  The study found that KT tools facilitated better understanding among stakeholders and improved interactions, which are crucial for enhancing mental health policies. The policy dialogue helped clarify different perspectives on deinstitutionalization and enabled participants to refine the evidence brief based on exhaustive discussions and feedback. |
| 1. Exploring the evolution of engagement between academic public health researchers and decision-makers: from initiation to dissolution | Jessani et al., 2020  US | The study population included 52 faculty members and 24 government decision-makers at city, state, federal and global levels | Qualitative study | Objective: to understand the drivers, enablers, and barriers to engagement between academic researchers and government decision-makers  Strategies:  The primary collaboration strategy used in the study revolves around developing and maintaining relationships through network building and leveraging social capital. This strategy emphasizes the importance of:   - Engagement through Established Networks: Utilizing existing networks and connections to initiate and foster relationships. - Co-production of Knowledge: Collaborating on research projects that involve both researchers and decision-makers in the process, thereby ensuring that the outputs are relevant and practically applicable. - Capacity Building Initiatives: Joint projects that aim to strengthen capacities on both sides, such as training sessions, workshops, and shared events. - Regular Communication and Feedback: Maintaining open lines of communication to ensure ongoing dialogue and adaptability to changing circumstances and needs.   This strategic approach highlights the dynamic and multifaceted nature of collaboration between academia and government, where both parties work together to address public health challenges effectively | Barriers   - Personnel Changes: Changes in key personnel within organizations can disrupt ongoing relationships and collaborations due to the loss of champions who understand and support the projects. - Transactional Relationships: Partnerships that become purely transactional, where the engagement is seen only as an exchange of services without deeper collaboration, can lead to dissolution. - Funding Constraints: Limited availability of funds can restrict the ability of parties to initiate or sustain engagement, especially in contexts requiring substantial resources. - Bureaucratic Challenges: The complexity of contractual agreements and procurement processes, especially when dealing with private universities, poses significant challenges. - Mismatched Priorities: Diverging priorities between academic institutions and government agencies can hinder effective collaboration, especially when the academic goals of publication and grant acquisition do not align with practical policy needs.   Facilitators   - Social Capital and Networks: Personal connections, alumni networks, and referrals play crucial roles in initiating and maintaining relationships. - Mutual Benefits: Collaborations that offer clear benefits to all parties involved tend to be more sustainable. These benefits include shared knowledge, capacity building, and enhanced decision-making. - Institutional Reputation: The reputation of the academic institution as a credible and reliable partner facilitates the establishment of new relationships. - Champions within Organizations: Having advocates or champions within the government or academic institutions who drive the relationships forward and maintain momentum. - Strategic Positioning and Alignment: Aligning the goals of the collaboration with the strategic interests and missions of the involved institutions enhances engagement and support.   Outcomes   - Drivers of Engagement: The study identified several key drivers for initiating and maintaining relationships, including decision-makers' research needs, access to unique resources, learning opportunities, and the personal and institutional reputation of academic researchers. - Facilitators and Barriers: Important facilitators for successful engagement included the existence of mutual benefits, social capital, institutional support, and the presence of champions or advocates within organizations. Conversely, barriers included funding constraints, bureaucratic challenges, personnel changes, and the transactional nature of some relationships that lacked deeper collaborative engagement. - Role of Social Capital: The study underscored the importance of social capital, such as personal connections and networks, which are crucial in initiating and sustaining these collaborative relationships. - Impact of Organizational Changes: Changes in personnel, especially the loss of champions who supported the collaboration, were noted as significant risks that could lead to the dissolution of partnerships. - Transactional vs. Transformative Relationships: A critical distinction was made between transactional relationships, which are often limited to specific deliverables, and transformative relationships, which are characterized by co-production of knowledge and joint capacity building, leading to more sustainable and impactful collaborations.   These results emphasize the complexity of academic-government collaborations in public health, highlighting the need for strategic planning, flexibility, and sustained effort to foster and maintain effective partnerships. The study also suggests that both academic institutions and government agencies can benefit from considering these dynamics when designing policies and practices to support collaboration  . |
| 1. How does embedded implementation research work? Examining core features through qualitative case studies in Latin America and the Caribbean | Varallyay et al., 2020  Latin America and the Caribbean | The participants included decision-makers and implementers in the health systems of Bolivia, Colombia, and the Dominican Republic. These were individuals involved at various levels of the health system, from policy-making to direct health service delivery. | The study utilized a qualitative, comparative case study approach. | Objective: to examine the core features of Embedded Implementation Research (EIR) and how these features influence evidence-to-action processes. It explored how EIR is operationalized, its effects, and the necessary supporting conditions across three projects in Bolivia, Colombia, and the Dominican Republic.  Strategies   - Regular Stakeholder Meetings: These meetings helped maintain engagement, gather feedback, and adapt the research process in real time to suit ground realities. - Workshops and Training Sessions: These were used to build capacity among stakeholders, ensuring that they could effectively contribute to and use research outputs. - Integration of Research and Practice: Efforts were made to align research activities closely with ongoing health programs, thereby increasing the likelihood that findings would be promptly and effectively utilized.   This study underscores the value of embedding research within the operational context of health programs and engaging directly with decision-makers and practitioners to ensure that research outputs are both relevant and immediately applicable. | Barriers:   - Resource Constraints: Limited funding and time were common challenges. - Cultural and Institutional Resistance: Differing organizational cultures and resistance to new methods within health systems posed significant obstacles.   Facilitators:   - Engagement of Stakeholders: Effective involvement of key stakeholders throughout the research process helped in mitigating resistance and enhancing the utility of findings. - Supportive Policy Environment: A supportive policy environment facilitated the application of research findings to real-world policy and program improvements.   Outcome:  The main results were organized around the four core EIR features:   - Central Involvement of Decision-Makers: The active participation of decision-makers in the research process enhanced the relevance and utilization of research findings. - Collaborative Research Partnerships: Strong, commitment-driven partnerships between researchers and practitioners facilitated effective research execution and application. - Positioning Research within Program Processes: This aspect was variably implemented across cases, with some indicating less necessity for tightly integrating research with ongoing program operations. - Research Focused on Implementation: Focusing on practical implementation challenges ensured that the research was directly applicable to improving program outcomes. |
| 1. Knowledge mobilisation in practice: an   Evaluation of the Australian Prevention  Partnership Centre | Haynes et al., 2020  Australian | Participants were stakeholders involved in the partnership, including policy-makers, practitioners, researchers, service users, and communities | A mixed-methods approach | Objective: to evaluate the operationalization and outcomes of knowledge mobilization strategies within the Australian Prevention Partnership Centre. This included exploring how cross-sector collaborative partnerships can strengthen research-informed policy and practice, particularly for chronic disease prevention  Strategies:   - Partnerships and Governance: Involvement of policy and practice partners in priority-setting, implementation planning, and governance. - Engagement Activities: Hosting interactive forums, funding collaborative research projects, and strategic communications. - Capacity and Skills Building: Conducting training for partners and facilitating cross-sector learning through workshops and forums. - Co-production Efforts: Promoting cross-sector investigator teams and adapting research projects to partners’ needs. - Knowledge Integration: Facilitating forums for synergy discovery across projects and synthesizing research findings for broader application. - Adaptive Learning: Implementing evaluation activities and utilizing feedback for continuous improvement.   This comprehensive approach helped the Center navigate the complexities of cross-sector partnerships and enhance the impact of its research on public health policy and practice | Barriers:   - Resource constraints, such as funding and time. - Cultural and institutional resistance to new methods. - Misalignments between research outcomes and policymaking needs.   Facilitators:   - Strong stakeholder engagement. - Effective use of structured knowledge mobilization tools. - Supportive policy environments that facilitated the application of research findings.   Outcome: The results indicated that the Center's produced research, resources, tools, and methods significantly impacted policy formation and funding. Policy-makers employed new methodologies that aided in designing, implementing, evaluating, and securing funding for policies and programs. The activities used to promote engagement, capacity building, and partnership formation generally yielded positive results, but there was room for improvement in co-production through enhanced shared decision-making. Additional efforts were necessary to operationalize knowledge integration and adaptive learning fully |
| 1. Leveraging Public Health Research to Inform State Legislative Policy   That Promotes Health for Children and Families | Tomayko et al.,  2019  USA | Participants in the Oregon Family Impact Seminar (OFIS) process include state legislators, state agency leaders, directors from research and social service organizations, and program officers from philanthropic foundations. | The OFIS operates through a systematic six-step process.  Strategic intervention | Objective: The primary objective of OFIS is to use public health research to inform state legislative policymaking, specifically targeting issues that affect children and families. The initiative focuses on bridging the communication gap between policymakers and researchers, providing a structured process to present synthesized research findings directly to state legislators.  The systematic 6-steps process include:  Strategies  (OFIS is a series of seminars aimed at facilitating communication between public health researchers and state legislators to influence policy. The process described is more about engaging key stakeholders and decision-makers in the state of Oregon, particularly through seminars held at or near the state legislature)  OFIS uses several collaboration strategies to ensure effective engagement with policymakers:   - Interdisciplinary planning committees to leverage diverse expertise and maintain continuity. - Direct engagement with legislators to align seminar topics with current legislative interests. - In-depth preparation of speakers to ensure clarity and relevance of presentations to legislative audiences. - Multiple points of contact with policymakers, including follow-up with research briefs to reinforce seminar content and encourage ongoing dialogue.   These structured interactions and the strategic planning of topics and discussions are designed to optimize the impact of public health research on state policy, particularly in areas affecting children and families. | Barriers and Facilitators  Key barriers include the inherent communication gaps and cultural differences between the academic and legislative communities. Facilitators include the strategic recruitment of legislative champions and the use of a nonpartisan, solution-oriented approach to discuss family-relevant public health issues.  Main Outcome  OFIS has successfully influenced policy changes, such as the increase in the Earned Income Tax Credit for parents with young children and the implementation of mandatory physical education for K-8 students. The seminars have also strengthened the relationships between researchers and legislators, improving the policy impact of future seminars |
| 1. Collaborative health research partnerships:   A survey of researcher and knowledge-user attitudes and perceptions | Sibbald et al.,  2019  Canada | The participants included researchers and various stakeholders such as health system managers, policymakers, and clinicians, who were involved in health research projects funded by the CIHR. | Quantitative research using online survey | Objective: to explore the attitudes and perceptions of both researchers and knowledge-users involved in collaborative health research partnerships. The goal was to assess how these partnerships impact the research process and outcomes, particularly in the context of Integrated Knowledge Translation (IKT) funded by the Canadian Institutes of Health Research (CIHR)  Strategies:  Based on the study's findings, several specific collaboration strategies were employed by participants:   - Structured Workshops and Seminars: These were used to bring together diverse stakeholders to discuss research developments, share insights, and integrate knowledge across disciplines. - Regular Meetings and Updates: Frequent meetings were crucial in maintaining momentum and ensuring that all parties were informed and engaged throughout the research process. - Joint Decision-Making Processes: Involving stakeholders in decision-making not only empowered participants but also ensured that diverse perspectives were considered in the research approach. - Use of Communication Technologies: To facilitate effective and efficient communication, various technologies were employed, including online collaboration tools, which helped bridge geographical distances and allowed for continuous interaction.   These strategies were instrumental in overcoming some of the barriers faced by the research teams and in capitalizing on the facilitators to enhance the effectiveness and impact of the collaborative research efforts. The approach highlighted the importance of planned, structured, and well-managed collaboration to handle the complexities of multi-stakeholder public health research | Barriers   - Resource Limitations: One of the main barriers identified was the limited resources available for conducting in-depth collaborative research. This included financial constraints, time limitations, and human resource restrictions. - Cultural and Organizational Differences: Differences in organizational cultures and operational priorities between institutions often hindered smooth collaboration. - Communication Gaps: Ineffective communication strategies sometimes lead to misunderstandings or misalignments of project goals among stakeholders.   Facilitators   - Strong Leadership and Clear Roles: Effective leadership and clearly defined roles within the research team facilitated smoother interactions and processes. Leaders who could navigate the complexities of multi-stakeholder environments were particularly valuable. - Established Protocols for Engagement: Protocols and frameworks that outlined how stakeholders should interact and collaborate throughout the research process helped in managing expectations and enhancing participation. - Regular Feedback Mechanisms: Systems set up to provide ongoing feedback allowed for timely adjustments to strategies and approaches, thus improving outcomes.   Outcome  The study provided several significant findings regarding the experiences of stakeholders in collaborative public health research:   - - Positive Impact of Collaboration: The results showed that when collaboration was effectively managed, it significantly improved research relevance and the application of findings in practice.   - Perceived Value: Stakeholders generally viewed the collaborative process positively, recognizing its potential to enhance the impact of public health research.   - Challenges in Integration: Despite the positive views, there were challenges in fully integrating knowledge from different stakeholders into the research process, which sometimes led to discrepancies in expectations and outcomes. |
| 1. How are evidence generation partnerships   Between researchers and policy-makers  Enacted in practice? A qualitative interview  Study | Williamson et al.,  2019  Australia | The participants included 18 key informants from New South Wales, Australia. This group was composed of researchers, policy and program developers as well as health system decision-makers. | Qualitative study design | Objective: to explore the enactment of evidence generation partnerships between researchers and policy-makers, identifying the reasons behind these collaborations, the common models of partnership, and the key components that lead to successful outcomes.  The collaboration strategies identified were it can be considered as facilitators more than strategy   - Developing shared aims and goals at the outset of projects. - Establishing clear governance structures and processes. - Ensuring continuous engagement and adequate funding. - Co-producing research to integrate diverse types of knowledge, thereby increasing the relevance and utility of the research outcomes.   This detailed examination of the enactment of evidence generation partnerships provides a comprehensive understanding of the dynamics involved in researcher-policy maker collaborations, highlighting the complexities and key strategies that can lead to successful outcomes. | Barriers and Facilitators  Barriers included differing priorities between researchers and policy-makers, issues with communication, and the challenges of aligning project timelines and outcomes with policy cycles. Facilitators of successful partnerships included a strong alignment around shared goals, mutual respect, clear communication, and well-structured governance processes.  Main outcome  The study found that both researcher-initiated and policy agency-initiated partnerships were common, with each having specific advantages. Policy-initiated partnerships were often deemed more likely to result in impactful outcomes due to their direct relevance to ongoing policy issues. Researcher-initiated projects, on the other hand, were valued for advancing scientific knowledge and meeting academic goals. The concept of co-production, where research is collaboratively developed from start to finish, was highlighted as a highly effective but challenging approach. |
| 1. Engaging Stakeholders, from Inception and Throughout the Study,   Is Good Research Practice to Promote use of Findings | Kalibala et al.,  2019  Low- and middle-income countries (LMICs) | The study involved multiple stakeholders including researchers, end data users (policy makers, program managers), and community stakeholders | The study adopted a systematic approach to research utilization | Objective: to actively promote the utilization of research findings in decision-making processes in low- and middle-income countries (LMICs). The study aimed to shift from passive dissemination of results to a more proactive facilitation of research utilization.  • The systematic approach to research utilization involved stakeholders from the inception of the research process through to the dissemination of findings. This approach included the use of a full-time knowledge use broker (RU Advisor) who provided technical assistance to study teams, facilitating stakeholder engagement at various phases of the research.  Strategy:   - Initial Engagement and Assessment:   Before starting any study, a thorough assessment was conducted to understand the local policy and stakeholder landscape. This included identifying key policymakers who could influence or benefit from the research findings.  • Policymakers were often included in Research Advisory Committees (RACs) or Technical Working Groups (TWGs) established for specific studies. These panels played a crucial role in shaping the research questions and ensuring the studies were aligned with policy needs.   - Continuous Involvement throughout research process:   • Input in Study Design: Policymakers provided input during the study design phase to ensure the research addressed relevant and practical policy questions. This was facilitated through structured engagements like workshops or meetings where study objectives and methodologies were discussed.  • Ongoing Consultations: Through continuous interactions facilitated by tools like the Stakeholder Engagement Matrix, researchers kept policymakers updated and involved in the research process. This tool helped in planning, documenting, and following up on engagements with policymakers.   - Capacity Building:   Training sessions were held for policymakers to enhance their ability to understand and utilize research findings effectively.   - Interpreting and Implementing Findings:   Policymakers collaborated in interpreting data and were crucial in translating research findings into actionable policy changes, facilitated by discussions and the development of action plans.   - Knowledge Brokers:   RU Advisors acted as liaisons between researchers and policymakers, ensuring effective communication and systematic engagement.   - Accessible Communication:   Research outputs were presented in accessible formats like policy briefs to ensure findings were clear and actionable for policymakers.  . | Barriers and Facilitators  Barriers: Common barriers included the traditionally linear perception of research impact, timing and opportunity constraints for using research, and stakeholders' responsiveness to research findings.  Facilitators: Facilitators included the establishment of a dedicated role for facilitating research utilization (RU Advisor), the creation of structured guides and tools for stakeholder engagement, and the proactive involvement of stakeholders in the research process.  Outcome  Although many of the studies were still ongoing at the time of reporting, early lessons indicated successful engagement of stakeholders throughout the research process. Preliminary findings were used to inform ongoing projects and policy adjustments, demonstrating the efficacy of active stakeholder engagement in promoting the use of research findings. |
| 1. Bridging the gap between research, policy, and   Practice: Lessons learned from academic–public  Partnerships in the CTSA network | Towfighi et al.,  2019  United state | The participants included seven Clinical and Translational Science Award (CTSA) hubs collaborating with city, county, and state healthcare and public health organizations. These included a wide range of stakeholders from academic institutions, public health departments, and healthcare systems across cities like Los Angeles, Chicago, Miami, and San Francisco. | The study employed a collaborative approach with monthly teleconferences among representatives from the CTSA hubs and public health/health system partners. | Objective: to bridge the gap between research, policy, and practice by fostering sustainable infrastructure that brings researchers, policymakers, practitioners, and communities together. This initiative aimed to reduce knowledge silos and enhance the translation of clinical research discoveries into improved population health and healthcare delivery.  Strategies:   - Engagement and Partnership Building: Engaging diverse stakeholders throughout the research process to ensure relevance and applicability of research efforts. - Knowledge Exchange and Dissemination: Employing strategies to ensure that findings are disseminated effectively and translated into practice. - Capacity Building and Training: Providing training and support to stakeholders to build capacity for effective participation in the research and policy-making process. - Integrated Approach: Using an integrated approach to research translation that involves stakeholders at all stages, from research design to implementation and evaluation. - Regular communication was facilitated through monthly teleconferences and ongoing collaborations | Barriers and Facilitators   - Barriers: Competing priorities, differing timelines, bureaucratic hurdles, and unstable funding. - Facilitators: Mutual benefits, strong leadership support, alignment with organizational priorities, clear roles, trust, effective communication, and adaptability.   Outcome:  The collaborations were successful in several areas:   - Aligning CTSA activities with local health system needs. - Promoting best practices and community-engaged research. - Narrowing the knowledge-to-practice gap through dissemination and implementation science |
| 1. Unknown makes unloved—A case study on improving   Integrated health and social care in the Netherlands using a  Participatory approach | Lette, et al.,  2019  Netherlands | Managers and professionals from home care organizations, integrated community care organizations, social care organizations, and general practice.  Policy officers from the municipality.  Representatives from regional advocacy organizations for older people. | A participatory case study design | Objective: to improve integrated health and social care in the Netherlands using a participatory approach. The study aimed to enhance collaboration between health and social care professionals to provide better care and support to older people living at home.  Strategies:   - Intervision meetings: Regular meetings focusing on reflection and mutual learning among professionals. - Workplace visits: Professionals visited and shadowed each other to better understand each other's roles and responsibilities. - Regular steering group meetings: These meetings involved managers and were aimed at identifying potential improvement areas, defining shared objectives, and developing improvement activities. | Facilitators: Safe and informal settings of meetings, personal relationships developed during the project, and the commitment of participating managers.  Barriers: Different organizational cultures and interests, a lack of ownership and accountability among managers, and external challenges such as staff shortages, time constraints, and privacy regulations  Outcome:   - Improvement activities enhanced communication and collaboration among professionals, establishing mutual understanding and trust. - Professionals reported that these activities improved their awareness of each other’s roles and responsibilities. - The participatory approach encouraged the development of partnerships and shared goals at both managerial and professional levels |
| 1. Collaborating with end-users in evidence synthesis: case studies for prevention in the first 2000 days | (Chung et al., 2024)Australia | Participants in the study included:  Researchers: From various Australian universities and research institutions.  Policymakers: State and federal health policymakers engaged through structured roundtables and meetings.  End-users: Including practitioners and caregivers who were involved in defining research questions and interpreting findings​ | The study employed a qualitative approach, focusing on two case studies that illustrate collaborative evidence synthesis efforts:  Case Study 1: Prevention in the First 2000 Days Knowledge Synthesis  • A collaboration established by the Collaboration for Enhanced Research Impact (CERI) involving researchers from Australian CREs, state and federal health policymakers, and science communication experts.  • The Prevention Centre managed the engagement process through policy roundtable meetings to establish evidence needs and guide research questions​​.  Case Study 2: The TOPCHILD (Transforming Obesity Prevention for CHILDren) Collaboration  • An international collaboration involving more than 100 researchers aimed at transforming obesity prevention in early childhood.  • The collaboration applied innovative evidence synthesis approaches, including individual participant data meta-analysis and prospective meta-analysis, to tailor findings to the needs of end-users​​. | Objective: to reflect on the experiences of undertaking collaborative evidence syntheses with end-users to inform policy and practice around preventive health in the early years of life (first 2000 days). The study aims to share key learnings to inform future collaborations in public health research, emphasizing the benefits of involving end-users in the evidence synthesis process​​.  Collaboration strategies:  1-Early and Genuine Partnerships: Establishing relationships with stakeholders early in the process, leveraging existing trusted relationships to ensure genuine partnerships.  2-Common Goals: Identifying and aligning common goals between researchers and end-users to ensure the relevance of the evidence synthesis.  3-Prioritizing Evidence Synthesis Aims: Ensuring the aims and objectives of the evidence synthesis are policy and practice relevant.  4-Transparent Communication: Maintaining open and two-way communication throughout the process to facilitate mutual understanding and trust​​. | Barriers to Collaboration   1. Time Constraints: The additional time required for collaborative processes, such as roundtable meetings and consensus-building, posed challenges. 2. Divergent Opinions: Managing differing opinions among stakeholders sometimes led to robust discussions, which required careful management to achieve consensus. 3. Complexity of Methods: Advanced evidence synthesis methods, such as individual participant data meta-analysis, required lengthy timeframes and additional resources​​.   Facilitators to Collaboration   1. Existing Relationships: Leveraging pre-existing relationships between researchers and policymakers facilitated smoother collaboration. 2. Stakeholder Commitment: Strong commitment from stakeholders, motivated by the relevance and applicability of the research, enhanced engagement. 3. Clear Governance and Leadership: Establishing clear governance structures and having responsive leadership teams helped in managing the collaborative process effectively​​.  - Outcome   The primary outcome of the collaborative approach was the production of evidence syntheses that were relevant and accessible to policymakers and other end-users. The study demonstrated that collaborative approaches to evidence synthesis can enhance the relevance and utility of research findings, leading to better-informed policy and practice decisions​​. |
| 1. Co‑creation of new knowledge: Good fortune or good management? | (Pearce et al., 2022)Australia | Researchers, third sector organization stakeholders (TSO) and funders | Case study design | Objective: to explore the processes and outcomes of co-creating new knowledge through participatory research involving multiple stakeholders. The study aims to document the events, critical factors influencing implementation, and the value of co-creation opportunities presented during the development of the Eclipse program, a psychoeducation group for people who have previously attempted suicide  The study followed a co-creation framework, involving four collaborative processes:   1. Co-Ideation:    - Generating ideas collaboratively. 2. Co-Design:    - Designing the program or policy and the research methods. 3. Co-Implementation:    - Implementing the program or policy according to agreed research methods. 4. Co-Evaluation:    - Collecting, analyzing, and interpreting data​​. | Barriers to Collaboration  1-Challenges with Trust Formation:  • Trust was integral and was facilitated by pre-existing relationships among researchers, stakeholders, and funders.  • Issues arose from a lack of familiarity and commitment in other sites not involved in the initial co-creation process​​.  2-Messiness of Co-Creation:  The inherent complexity and “messiness” of co-creation required adaptation and flexibility from all parties involved​​.  Facilitators to Collaboration  1-Complementary Expertise and Skills:  Researchers and stakeholders brought complementary skills that enhanced collaboration.  2-Commitment and Flexibility:  • Continued commitment despite critical events (e.g., COVID-19, changes in key staff, loss of funding).  • Adaptation to changes, such as moving to online delivery during the pandemic​​.  3-Regular Communication:  • Frequent meetings and correspondence ensured continuous collaboration and adaptation.  • Sharing explicit and tacit knowledge led to greater collaborative reciprocity​​.  Outcome  The primary outcome of the collaborative approach was the successful implementation of the co-creation framework, highlighting the importance of trust, good governance, and good fortune. The study identified new areas for integration into the co-creation framework, including pre-co-creation stages and spin-off opportunities . |
| 1. Integrated Knowledge Translation n Non-Communicable Disease Research in Sub-Saharan Africa: A Comparison of Systematic and Ad Hoc Stakeholder Engagement | (Mpando et al., 2021)Sub-Saharan Africa | • Country Teams: Researchers and stakeholders from five Sub-Saharan African countries participated, each team tailoring the general IKT framework to their specific context​​.  • Stakeholders: This included government officials, policymakers, community health workers, and other relevant stakeholders in the field of non-communicable diseases (NCDs) in each participating country​​.  • Research Subjects: The study did not specify individual subjects as it focused on organizational and systemic stakeholder engagement rather than individual clinical subjects​​. | A cross-sectional study design  Using a self-administered semi-structured questionnaire.  Data sources included:  1-Informal Document Review: CEBHA+ country-specific IKT strategies, IKT team meeting minutes, and activity reports were reviewed.  2-Semi-Structured Survey: A survey of IKT implementers was conducted to elicit country-specific experiences on the implementation and adaptation of IKT strategies. The survey requested details of stakeholder engagement strategies, successful implementations, instances of ad hoc engagement, and the benefits and disadvantages of both approaches​​. | Objective:  To explore the experiences of researchers engaged in the CEBHA+ Integrated Knowledge Translation (IKT) approach across five African countries. The goal was to compare systematic and ad hoc stakeholder engagement strategies and their effectiveness in facilitating the uptake of NCD research​​  Strategy of Collaboration:  The collaboration strategy involved several key components:  1-Stakeholder Mapping and Engagement:  Each country team plotted stakeholders on a power/interest grid to prioritize engagement and developed detailed engagement strategies, specifying the main message, the messenger, the medium, and uptake indicators.  Regular meetings were held with IKT focal points from each site to discuss implementation and experiences​​.  2-Systematic vs. Ad Hoc Engagement:  Systematic Engagement: This approach involved deliberate, planned stakeholder engagement to ensure focused and intentional collaboration. It allowed for structured interactions and was beneficial in the early stages of the project.  Ad Hoc Engagement: Due to contextual changes, including the COVID-19 pandemic, responsive engagement with decision-makers and other stakeholders became necessary. This mode of engagement was more reactive and covered topics beyond the original project focus​​.  3- Country specific capacity Building and Joint Activities:  Ethiopia: Engaged 18 stakeholders through in-person meetings, telephone calls, and emails. Focused on capacity-building activities like trainings and workshops.  Malawi: Identified 14 priority stakeholders, including policymakers and NCD advocacy groups. Engagement methods included in-person meetings, workshops, emails, and phone calls.  Rwanda: Identified 18 priority stakeholders, including policymakers, health practitioners, and civil society organizations. Engagement methods included in-person and online meetings, emails, phone calls, and social media interactions.  South Africa: Identified 20 stakeholders, eight of which were prioritized. Engagement focused on establishing and enhancing partnerships, promoting research collaboration, and influencing NCD policy and practice.  Uganda: Identified priority stakeholders, including policymakers and law enforcement. Engagement focused on research collaboration, agenda setting, and increasing research awareness​​. | Barriers to Collaboration:   - Staff Turnover: High turnover within stakeholder institutions, especially government bodies, disrupted continuity in Ethiopia, Uganda, and Rwanda​​. - Budgeting Challenges: Inconsistent resources for ad hoc activities caused potential budgeting issues​​. - Time Constraints and Establishing Relationships: Time constraints and failure to establish some intended relationships were noted as significant challenges​​.   Facilitators of Collaboration:   - Pre-existing Relationships: Leveraging existing professional relationships facilitated engagement in several countries​​. - Stakeholder Commitment: Commitment from stakeholders motivated their interaction with researchers, as seen in Uganda with road safety and in South Africa with the political prioritization of NCDs​​. - Resource Allocation: Dedicated resources, both financial and human, were crucial for stakeholder engagement​​.   Main Outcomes of the Collaboration Approach:   - Improved Relationships: Establishing and enhancing relationships with stakeholders was a key outcome, leading to better engagement and collaboration​​. - Identification of Gaps: The IKT approach helped identify research and capacity gaps, particularly around evidence-based policymaking​​. - Enabling Environment for Research Uptake: The systematic IKT approach created a conducive environment for research uptake into policy and practice, resulting in better science and more relevant findings. For example, it led to the development of an electronic data system for road traffic crashes in Uganda​​. - Policy Influence: There was evidence of IKT influencing policy and practice, such as the rapid evidence syntheses requested by the South African National Department of Health during the COVID-19 pandemic​​. |
